# Sex‐Specific Treatment Effects After Primary Percutaneous Intervention: A Study on Coronary Blood Flow and Delay to Hospital Presentation

**DOI:** 10.1161/JAHA.118.011190

**Published:** 2019-02-15

**Authors:** Edina Cenko, Mihaela van der Schaar, Jinsung Yoon, Sasko Kedev, Marija Valvukis, Zorana Vasiljevic, Milika Ašanin, Davor Miličić, Olivia Manfrini, Lina Badimon, Raffaele Bugiardini

**Affiliations:** ^1^ Department of Experimental, Diagnostic and Specialty Medicine University of Bologna Bologna Italy; ^2^ University of Cambridge Cambridge United Kingdom; ^3^ Department of Electrical and Computer Engineering University of California, Los Angeles Los Angeles CA; ^4^ Medical Faculty University Clinic of Cardiology University “Ss Cyril and Methodius” Skopje Macedonia; ^5^ School of Medicine University of Belgrade Belgrade Serbia; ^6^ Department for Cardiovascular Diseases University Hospital Center Zagreb University of Zagreb Zagreb Croatia; ^7^ Cardiovascular Program (ICCC) IR‐Hospital de la Santa Creu i Sant Pau CiberCV‐Institute Carlos III Autonomous University of Barcelona Barcelona Spain; ^8^ Department of Cardiology Clinical Centre of Serbia Belgrade Serbia

**Keywords:** coronary blood flow, mortality, sex differences, ST‐segment–elevation myocardial infarction, Quality and Outcomes, Mortality/Survival, Women

## Abstract

**Background:**

We hypothesized that female sex is a treatment effect modifier of blood flow and related 30‐day mortality after primary percutaneous coronary intervention (PCI) for ST‐segment–elevation myocardial infarction and that the magnitude of the effect on outcomes differs depending on delay to hospital presentation.

**Methods and Results:**

We identified 2596 patients enrolled in the ISACS‐TC (International Survey of Acute Coronary Syndromes in Transitional Countries) registry from 2010 to 2016. Primary outcome was the occurrence of 30‐day mortality. Key secondary outcome was the rate of suboptimal post‐PCI Thrombolysis in Myocardial Infarction (TIMI; flow grade 0–2). Multivariate logistic regression and inverse probability of treatment weighted models were adjusted for baseline clinical covariates. We characterized patient outcomes associated with a delay from symptom onset to hospital presentation of ≤120 minutes. In multivariable regression models, female sex was associated with postprocedural TIMI flow grade 0 to 2 (odds ratio [OR], 1.68; 95% CI, 1.15–2.44) and higher mortality (OR, 1.72; 95% CI, 1.02–2.90). Using inverse probability of treatment weighting, 30‐day mortality was higher in women compared with men (4.8% versus 2.5%; OR, 2.00; 95% CI, 1.27–3.15). Likewise, we found a significant sex difference in post‐PCI TIMI flow grade 0 to 2 (8.8% versus 5.0%; OR, 1.83; 95% CI, 1.31–2.56). The sex gap in mortality was no longer significant for patients having hospital presentation of ≤120 minutes (OR, 1.28; 95% CI, 0.35–4.69). Sex difference in post‐PCI TIMI flow grade was consistent regardless of time to hospital presentation.

**Conclusions:**

Delay to hospital presentation and suboptimal post‐PCI TIMI flow grade are variables independently associated with excess mortality in women, suggesting complementary mechanisms of reduced survival.

**Clinical Trial Registration:**

URL: http://www.clinicaltrials.gov. Unique identifier: NCT01218776.

## Abstract 2

See Editorial by Pande and Jacobs

**Clinical Perspective**

**deos Is New?**

- In the current study, compared with men, postprocedural Thrombolysis in Myocardial Infarction flow grade rates were lower and 30‐day mortality was higher in women.
- The distribution of covariates was similar between women and men.
- Delay to hospital presentation also modified comparative effectiveness between sexes because the sex gap in mortality was no longer significant for patients presenting to hospital within 120 minutes.

**What Are the Clinical Implications?**

- Differences between men and women in post primary percutaneous coronary intervention mortality rates exist even when women and men are balanced in terms of baseline characteristics, treatment, and time to hospital presentation.
- Delay to hospital presentation and suboptimal postprocedural Thrombolysis in Myocardial Infarction flow grade are variables independently associated with excess mortality in women, suggesting complementary mechanisms of reduced survival.
- Sex as a biological variable should be factored into research designs, analyses, and reporting in human studies.

## Introduction

Possible sex‐based differences with respect to atherosclerotic disease have become a subject of intense research and debate. Atherosclerosis tends to affect men and women differently and at different times in their lives. One of the most intriguing findings is that women presenting with ST‐segment–elevation myocardial infarction (STEMI) die more often than men irrespective of reperfusion modalities.^1^, ^2^, ^3^, ^4^, ^5^, ^6^, ^7^ These findings have raised the physician's interest in finding out more about the reasons for this large sex gap. Yet, such information is missing because studies on pathophysiological characteristics of ischemic heart disease have mainly focused on women with the exclusion or underrepresentation of men.^8^

Coronary blood flow is a major determinant of patients’ prognosis.^9^ Restoration of blood flow after recanalization of the infarct‐related artery is commonly quantified with the Thrombolysis in Myocardial Infarction (TIMI) grading system.^10^ Failure to account for sex differences in coronary blood flow after primary percutaneous coronary intervention (PCI) could explain contradictory findings on the rates of mortality among women and men.^11^ Abnormal coronary reactivity and microvascular dysfunction are more prevalent among women^12^ and may be associated with lower TIMI flow grades and adverse outcomes. In addition, myocardial regions subjected to prolonged ischemia exhibit loss of microvascular integrity with endothelial swelling and edema.^13^ The extent of lesion depends on the duration of ischemia, and women have longer delays to hospital presentation compared with men.^4^, ^5^, ^14^

The purpose of our study was to characterize clinical and angiographic variables potentially associated with suboptimal coronary blood flow and their association with outcomes in a large cohort of patients undergoing primary PCI. The primary hypothesis was that post‐PCI coronary blood flow might differ for men and women and may partially account for increased 30‐day mortality among women. We also hypothesized that the magnitude of the effect on outcomes differs depending on delay to hospital presentation.

## Methods

### Study Population

The authors declare that all supporting data are available within the article and its supplemental material.

The ISACS‐TC (International Survey of Acute Coronary Syndromes in Transitional Countries; ClinicalTrials.gov: NCT01218776) registry is a large, prospective, multicenter cohort study designed to record clinical characteristics, treatments, and clinical outcomes for patients with acute coronary syndrome.^5^, ^15^ The ISACS‐TC registry enrolled 5590 patients who underwent primary PCI for STEMI within 12 hours from symptom onset between January 2010 and January 2016. The designated physician collected the registry data at the time of clinical assessment. Patients were asked about the quality of their symptoms. All patients presented with chest pain or equivalent symptoms, such as dyspnea and fatigue. Data on coronary hemodynamics were available in 2651 of the 5590 patients. In the remaining patients, the ISACS‐TC registry generated reports with summarizing information on PCI procedural success without any further detail. Among the 2651 patients with data on coronary hemodynamics, those who had an unknown time of symptom onset (n=55) were excluded, giving a final study population of 2596 patients (Figure). Patients who were transferred from a PCI noncapable hospital were included in this analysis. Time of symptom onset relied on patient recall and required documentation by each hospital participating in this registry. Delay time from symptom onset to hospital presentation included door‐in to door‐out time, defined as the duration of time from arrival to discharge at the first referral hospital, and transfer time to a STEMI receiving hospital for primary PCI. Because delay times are a continuous variable, we characterized time to reperfusion and patient outcomes associated with a time from symptom onset to hospital presentation of ≤120 minutes and door‐to‐balloon time of ≤90 minutes according to prior work.^4^, ^14^ The local research ethics committee from each hospital approved the study. The data‐coordinating center has been established at the University of Bologna (Bologna, Italy). The study was approved by the local research ethics committee from each hospital. Because patient information was collected anonymously, institutional review boards waived the need for individual informed consent.

**Figure 1:**
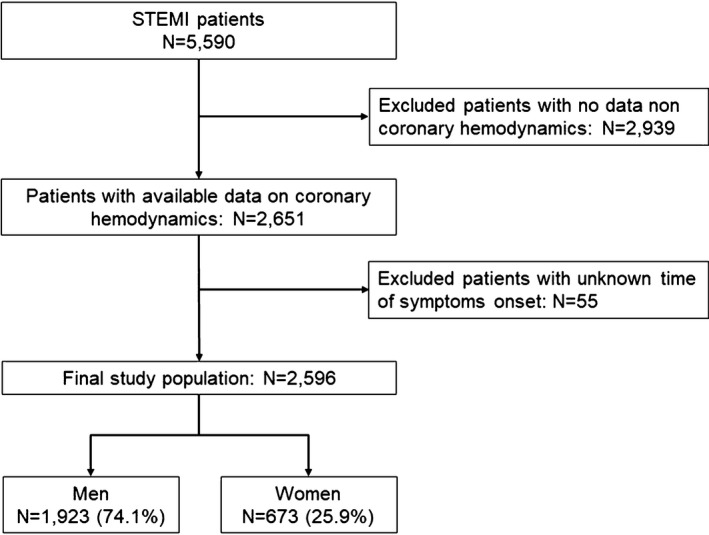
Flow diagram. STEMI indicates ST‐segment–elevation myocardial infarction.

### Outcomes

The primary outcome was the incidence of 30‐day all‐cause mortality. Key secondary outcome was the incidence of suboptimal TIMI flow grade 0 to 2 after PCI. We also studied the likelihood of having ischemic stroke and bleeding complications. Ischemic stroke was diagnosed on the basis of an imaging study. Bleeding complications were defined as previously reported by the TIMI definitions.^16^ Decreases in hemoglobin ≥5 g/dL occurring during hospitalization or intracranial hemorrhage classified bleeding as major. The use of medications given at hospital admission was also noted.

### TIMI Flow Grading

The TIMI flow of the infarct artery was estimated before and after completion of PCI, according to 4 grades of flow, as previously described.^10^ In brief, grade 0 perfusion is no antegrade flow beyond the point of occlusion; grade 1 is faint antegrade coronary flow beyond the occlusion with incomplete filling of the distal coronary bed; grade 2 is complete but delayed filling of distal coronary bed; and grade 3 is normal flow with complete filling of the distal bed at a normal rate. Patients were divided into 2 groups on the basis of the postprocedural TIMI flow in the ischemia‐related artery: TIMI grades ≤2 (no reflow) and TIMI grade 3. The definition of no reflow required the absence of coronary dissection or significant (≥25%) residual stenosis that could have caused a decrease in coronary blood flow.^17^

### Statistical Analysis

Patient and procedure characteristics were examined according to sex. Statistical testing was performed with the use of the Pearson's χ² test for categorical variables and the 2‐sample *t* test for continuous variables. We had complete data on time from symptom onset to PCI and mortality. Some patients had missing data on other variables. We imputed the missing values of the clinical variables whose missing rate was <10% using IVEWARE software.^18^ Only 1 variable, Killip class, had missing rates that exceeded 10%. We used k‐nearest neighbor algorithms as imputation method^19^, ^20^, ^21^ (Data S1). Estimates of the odds ratios (ORs) and associated 95% CIs were obtained with the use of multivariable logistic regressions. Fixed covariates included demographic information and baseline clinical characteristics (Tables 1 and 2). We stabilized weights to reduce the variability and ensure that the estimated treatment effect remains balanced.^22^ Weighted *t* tests and weighted χ^2^ tests were used in the inverse probability of treatment weighting (IPTW) analyses to compare continuous or categorical variables in women and men (Data S1). Furthermore, to assess significant heterogeneity of outcomes in function of sex and delay from symptom onset to hospital presentation, we made statistical comparisons across 2 delay cohorts (<120 and ≥120 minutes). We used IPTW because it is the simplest method that adjusts for the confounding effect of time‐varying covariates.^23^ Multivariable‐adjusted regression was inadequate in controlling time‐varying confounding^24^ and was not used for this task (Data S1).

**Table 1:** Baseline Characteristics of Patients With STEMI Sorted by Sex

Characteristics	Women (N=673)	Men (N=1923)	*P* Value
Age, mean±SD, y	64.8±11.5	58.8±11.1	<0.001
Cardiovascular risk factors, n (%)			
Family history of CAD	224 (33.3)	595 (30.9)	0.265
Diabetes mellitus	163 (24.2)	392 (20.4)	0.043
Hypertension	500 (74.3)	1198 (62.3)	<0.001
Hypercholesterolemia	340 (50.5)	838 (43.6)	0.002
Current smoking	278 (41.3)	1023 (53.2)	<0.001
Former smoking	45 (6.7)	286 (14.9)	<0.001
Previous cardiovascular disease, n (%)			
Previous angina pectoris	160 (23.8)	354 (18.4)	0.004
Previous myocardial infarction	36 (5.3)	149 (7.7)	0.024
Previous PCI	12 (1.8)	88 (4.6)	<0.001
Previous CABG	0 (0.0)	11 (0.6)	0.001
Peripheral artery disease	5 (0.7)	27 (1.4)	0.121
Previous heart failure	38 (5.6)	95 (4.9)	0.488
Previous stroke	16 (2.4)	46 (2.4)	0.983
Clinical presentation			
ST‐segment elevation in anterior leads, n (%)	232 (34.5)	745 (38.7)	0.047
Killip class ≥2, n (%)	100 (14.9)	215 (11.2)	0.018
Systolic blood pressure at baseline, mean±SD, mm Hg	134.6±26.6	136.6±24.5	0.094
Heart rate at baseline, mean±SD, beats/min	79.3±17.6	79.4±17.0	0.854
Serum creatinine at baseline, mean±SD, μmol/L	79.0±44.7	94.4±52.3	<0.001
Medications administered within 24 h from admission, n (%)			
Aspirin	667 (99.1)	1914 (99.5)	0.283
Clopidogrel	663 (98.5)	1856 (96.5)	0.001
Unfractionated heparin	377 (56.0)	1118 (58.1)	0.340
Low‐molecular‐weight heparins	318 (47.3)	933 (48.5)	0.571
Heparins (overall)	627 (93.2)	1809 (94.1)	0.415
Glycoprotein IIb/IIIa inhibitor	121 (18.0)	363 (18.9)	0.604
Outcomes			
30‐d Mortality, n (%)	40 (5.9)	45 (2.3)	<0.001
30‐d Mortality, OR (95% CI)	2.64 (1.66–4.17)		<0.001
TIMI flow grade ≤2, n (%)	54 (8.0)	91 (4.7)	0.004
TIMI flow grade ≤2, OR (95% CI)	1.76 (1.21–2.52)		0.002

CABG indicates coronary artery bypass graft; CAD, coronary artery disease; OR, odds radio; PCI, percutaneous coronary intervention; STEMI, ST‐segment–elevation myocardial infarction; TIMI, Thrombolysis in Myocardial Infarction.

**Table 2:** IPTW: Women Versus Men

Characteristics	Women (n=673)	Men (n=1923)	*P* Value
Age, mean±SD, y	61.8±11.6	60.3±11.4	0.003
Cardiovascular risk factors, %			
Family history of CAD	34.9	31.9	0.153
Diabetes mellitus	22.5	21.2	0.480
Hypertension	68.4	65.0	0.109
Hypercholesterolemia	46.6	44.0	0.243
Current smoking	47.9	50.2	0.304
Former smoking	9.5	12.6	0.031
Previous cardiovascular disease, %			
Previous angina pectoris	20.7	19.4	0.466
Previous myocardial infarction	5.6	7.1	0.180
Previous PCI	1.9	3.9	0.013
Previous CABG	0	0.4	0.112
Peripheral artery disease	0.7	1.2	0.286
Previous heart failure	5.2	5.1	0.919
Previous stroke	3.0	2.4	0.398
Clinical presentation at admission			
ST‐segment elevation in anterior leads, %	37.4	37.7	0.890
Killip class ≥2, %	19.2	19.3	0.954
Systolic blood pressure at baseline, mean±SD, mm Hg	134.8±27.6	136.0±24.3	0.324
Heart rate at baseline, mean±SD, beats/min	79.0±17.2	79.2±17.4	0.809
Serum creatinine at baseline, mean±SD, μmol/L	77.8±28.2	91.7±46.4	<0.0001
Outcomes			
30‐d Mortality, %	4.8	2.5	0.0024
30‐d Mortality, OR (95% CI)	2.00 (1.27–3.15)		0.0029
TIMI flow grade ≤2, %	8.8	5.0	0.0003
TIMI flow grade ≤2, OR (95% CI)	1.83 (1.31–2.56)		0.0004

CABG indicates coronary artery bypass graft; CAD, coronary artery disease; IPTW, inverse probability of treatment weighting; OR, odds radio; PCI, percutaneous coronary intervention; TIMI, Thrombolysis in Myocardial Infarction.

## Results

Similarities and differences between women and men excluded from the present analysis are shown in Table S1. Among the 2596 patients enrolled in the study, there were 1923 men (74%) and 673 women (26%) (Table 1). There was an imbalance of clinical covariates between women and men, as shown by the optimized regression coefficient (β) and constant term (α) for the logistic regression (Table S2). Women presented at an older age than men. They were more likely to have diabetes mellitus, hypertension, and congestive heart failure, but less likely to have a history of smoking. Crude mortality rates among women and men were 5.9% versus 2.3%. Angiograms revealed 8.1% of women and 4% of men with post‐PCI TIMI flow grade 0 to 2.

### Angiographic and Procedural Characteristics

Procedural characteristics stratified by sex are illustrated in Table S3. Women and men had similar rates of preprocedural TIMI flow grade 3 (26.4% versus 31.1%; *P*=0.13). There was no sex difference in frequency of multivessel disease, acute vessel closure, acute thrombosis, and bifurcation lesions. Bleeding complications and ischemic strokes occurred infrequently and were not significantly different between the 2 groups.

### Balancing Covariates: Descriptive Baseline Comparison

Standard parametric adjustment by regression could be sensitive to model misspecification when groups differ greatly, as we observed in our population for women versus men. We, therefore, balanced the distribution of covariates using a nonparametric balancing strategy by weighting (IPTW). The 2 groups (women and men) were well balanced (Table 2). The primary outcome of death occurred in 4.8% of women compared with 2.5% of men (reference, men; OR, 2.00; 95% CI, 1.27–3.15). Moreover, we found a significant sex difference in the secondary outcome of post‐PCI TIMI flow grade ≤2 (reference, men; OR, 1.83; 95% CI, 1.31–2.56). To determine the relation between sex and severe no reflow complicating primary PCI, we excluded patients who had TIMI flow grade 2. The sex difference in the incidence of TIMI flow grade ≤1 was similar (OR, 1.51; 95% CI, 1.02–2.26) to that seen for the entire cohort (TIMI flow grade ≤2) (Table S4).

### Multivariable Analysis of Baseline Clinical Factors Associated With Outcomes

To reinforce our data, we also estimated the multivariable‐adjusted effect of PCI on TIMI flow grade 0 to 2 and 30‐day mortality. Although the odds of a multivariable‐adjusted regression cannot calculate the absolute risk, female sex was still a predictor variable for the association with post‐procedural TIMI flow grade 0 to 2 (OR, 1.68; 95% CI, 1.15–2.44) (Table S5) and 30‐day mortality (OR, 1.72; 95% CI, 1.02– 2.90) (Table S6). It follows that linear regression adjustment resulted in conclusions similar to those obtained using IPTW methods.

### Delay to Hospital Presentation

Median time from symptom onset to hospital presentation was longer for women (280 versus 240 minutes), with only 23.2% of women versus 29.1% of men having a <120‐minute delay from symptom onset to admission (*P*=0.002) (Table S7). There were no differences between sexes in median door‐to‐balloon time (40 versus 38 minutes). The unadjusted risk of mortality increased with increasing time from symptom onset to hospital presentation (Figure Table S1). We therefore explored whether female sex could be a contributing factor that increases the risk of mortality when hospital presentation times were comparable among women and men.

### Sex Differences in Outcomes in Function of Delay to Hospital Presentation

We assessed how differences in time to hospital presentation could translate into sex‐specific PCI outcomes using IPTW (Table 3). For delayed time to hospital presentation ≥120‐minute benchmark, the mortality rates were 5.5% in women and 2.8% in men. When women and men presented to hospital within 120 minutes, their outcomes were equally positive (2.0% versus 1.6%). On the contrary, the incidence of post‐PCI TIMI flow grade ≤2 rate was higher in women compared with men regardless of time to hospital presentation: 9.4% versus 6.3% in patients with delay to hospital presentation >120 minutes and 7.9% versus 1.6% in those with delay <120 minutes.

**Table 3:** IPTW: Women Versus Men Sorted by Delay Cohorts

Variable	Timely (<120‐min) PCI			Delayed (≥120‐min) PCI		
	Women (n=156)	Men (n=560)	*P* Value	Women (n=517)	Men (n=1363)	*P* Value
Age, mean±SD, y	61.2±11.6	58.2±11.1	0.003	62.1±11.5	61.1±11.4	0.093
Cardiovascular risk factors, %						
Family history of CAD	34.3	34.5	0.963	33.9	31.0	0.228
Diabetes mellitus	17.7	16.3	0.678	23.8	23.1	0.748
Hypertension	68.7	59.9	0.045	69.3	66.9	0.321
Hypercholesterolemia	45.2	43.6	0.722	48.0	44.3	0.150
Current smoking	58.2	56.7	0.738	44.4	47.6	0.214
Former smoking	4.0	10.6	0.011	11.2	13.4	0.202
Previous cardiovascular disease, %						
Previous angina pectoris	20.0	17.6	0.491	20.9	20.1	0.701
Previous myocardial infarction	7.0	9.4	0.351	6.0	6.4	0.749
Previous PCI	3.1	6.1	0.145	1.6	3.0	0.089
Previous CABG	0	0.3	0.4813	0	0.5	0.101
Peripheral artery disease	0.8	0.9	0.9041	0.7	1.4	0.217
Previous heart failure	6.8	4.0	0.140	5.4	5.6	0.866
Previous stroke	4.1	1.9	0.112	2.8	2.7	0.905
Clinical presentation at admission						
ST‐segment elevation in anterior leads, %	42.4	42.7	0.946	34.9	35.9	0.686
Killip class ≥2, %	15.6	13.7	0.547	20.2	21.5	0.538
Systolic blood pressure at baseline, mean±SD, mm Hg	137.2±26.4	135.7±23.7	0.503	134.9±27.6	136.2±24.5	0.354
Heart rate at baseline, mean±SD, beats/min	77.1±13.5	77.8±169	0.669	79.1±17.6	79.7±17.5	0.475
Serum creatinine at baseline, mean±SD, μmol/L	72.8±19.4	90.7±35.4	<0.0001	78.2±28.8	92.1±50.2	<0.001
Outcomes						
30‐d Mortality, %	2.0	1.6	0.713	5.5	2.8	0.004
30‐d Mortality, OR (95% CI)	1.28 (0.35–4.69)		0.714	2.03 (1.23–3.33)		0.005
TIMI flow grade ≤2, %	7.9	1.7	0.0001	9.4	6.3	0.02
TIMI flow grade ≤2, OR (95% CI)	4.82 (2.04–11.41)		0.0003	1.54 (1.07–2.23)		0.021

CABG indicates coronary artery bypass graft; CAD, coronary artery disease; IPTW, inverse probability of treatment weighting; OR, odds radio; PCI, percutaneous coronary intervention; TIMI, Thrombolysis in Myocardial Infarction.

### Delay to Treatment and Mortality in “Flow Benefiters”

We wanted to create a challenge in defining the effect of delay to hospital presentation on the primary outcome of 30‐day mortality independently of the occurrence of suboptimal blood flow (grade 0–2). We, therefore, restricted our analysis to patients who fully benefited from PCI intervention (TIMI flow grade 3) (Table 4). The results were consistent with, although smaller in effect size than, those seen in the overall population. There were sex differences for late STEMI presentations (delay to hospital presentation ≥120 minutes), with women having worse outcomes (3.9% versus 2.0%; OR, 1.94; 95% CI, 1.06–3.57). In contrast, there was no significant difference in the rates of death in women and men with timely (<120‐minute) hospital presentation (1.1% versus 1.4%; OR, 0.74; 95% CI, 0.13–4.26).

**Table 4:** IPTW: PCI Treatment Effect on 30‐Day Mortality in “Flow Benefiters”

Characteristics	Timely (<120‐min) PCI			Delayed (≥120‐min) PCI		
	Women (N=143)	Men (N=549)	*P* Value	Women (N=469)	Men (N=1278)	*P* Value
Age, mean±SD, y	61.7±11.1	57.9±11.0	0.0003	62.3±11.7	60.9±11.4	0.023
Cardiovascular risk factors, %						
Family history of CAD	33.6	35.0	0.754	34.4	31.7	0.285
Diabetes mellitus	20.1	16.1	0.256	24.8	23.2	0.485
Hypertension	69.2	59.5	0.033	69.7	66.6	0.220
Hypercholesterolemia	45.0	43.6	0.764	48.3	44.2	0.127
Current smoking	59.0	57.1	0.683	43.9	47.8	0.148
Former smoking	4.7	10.5	0.033	10.9	13.3	0.181
Previous cardiovascular disease, %						
Previous angina pectoris	21.0	17.3	0.306	21.1	19.8	0.548
Previous myocardial infarction	7.5	9.9	0.382	5.2	6.3	0.392
Previous PCI	0.3	6.3	0.126	1.5	3.2	0.052
Previous CABG	0	0.3	0.507	0	0.5	0.132
Peripheral artery disease	0.8	0.9	0.909	1.0	1.1	0.855
Previous heart failure	7.5	4.1	0.089	5.2	5.7	0.686
Previous stroke	3.6	1.7	0.157	3.3	2.7	0.505
Clinical presentation at admission						
ST‐segment elevation in anterior leads, %	39.0	41.5	0.588	33.8	34.6	0.755
Killip class ≥2, %	15.1	13.0	0.512	19.4	19.8	0.852
Systolic blood pressure at baseline, mean±SD, mm Hg	137.2±28.2	135.8±23.5	0.555	134.8±28.0	136.6±24.4	0.210
Heart rate at baseline, mean±SD, beats/min	76.6±13.2	77.4±16.5	0.616	79.5±18.2	79.6±17.3	0.911
Serum creatinine at baseline, mean±SD, μmol/L	73.3±19.2	89.8±33.8	<0.001	79.0±28.8	90.2±45.9	<0.001
Outcomes						
30‐d Mortality, %	1.1	1.4	0.732	3.9	2.0	0.029
30‐d Mortality, OR (95% CI)	0.74 (0.13–4.26)		0.732	1.94 (1.06–3.57)		0.032

CABG indicates coronary artery bypass graft; CAD, coronary artery disease; IPTW, inverse probability of treatment weighting; OR, odds radio; PCI, percutaneous coronary intervention.

### Delay to Treatment and Mortality in Patients With TIMI Flow Grade 0 to 2

Next, we focused on patients who did not benefit from PCI intervention (TIMI flow grade 0–2). Figure S2 summarizes the relationship between delay to hospital presentation and mortality in patients with post‐PCI TIMI flow grades 0 to 2. The incidence of post‐PCI TIMI flow grades 0 to 2 in the overall population was 8.0% in women and 4.7% in men (Table 1). The unadjusted 30‐day mortality rates associated with post‐PCI TIMI flow grades 0 to 2 were 2.5% in women and 0.5% in men. Sex differences in patients with early, within 120 minutes, hospital presentations were comparable to those of the overall population (Table 3). There was a large difference in the incidence of post‐PCI TIMI flow grades 0 to 2 between women and men (7.9% versus 1.7%). Sex differences were less pronounced in late hospital presentations, in which the incidence of post‐PCI TIMI flow grades 0 to 2 was 9.4% in women and 6.3% in men. Unadjusted mortality rates in patients with TIMI flow grades 0 to 2 were >2‐fold higher in women compared with men in either early or late hospital presentation (28.6% versus 10.0% and 31.9% versus 11.1%, respectively). TIMI flow grades 0 to 2 remained a correlate of higher 30‐day mortality in women versus men even after removing confounding by IPTW (Tables S8 and S9).

### Age Groups and TIMI Flow Grade 0 to 2

We investigated the role of sex and age on TIMI flow by stratifying patients into 2 groups: aged <60 and ≥60 years. TIMI flow grades 0 to 2 remained an independent correlate of female sex only in patients aged ≥60 years (OR, 1.56; 95% CI, 1.08–2.26) (Tables S10 and S11).

## Discussion

Sex differences in mortality can be seen only in patients with STEMI.^25^, ^26^, ^27^, ^28^, ^29^, ^30^, ^31^, ^32^, ^33^ Many of the differences in outcomes have been attributed to sex differences in treatment because women present to hospital later than men and are less likely to undergo primary PCI.^30^, ^31^ There are few studies addressing the issue of sex differences in outcome after primary PCI.^1^, ^2^, ^3^, ^4^, ^5^, ^6^, ^7^, ^32^, ^34^, ^35^, ^36^, ^37^ Yet, most of these studies still documented persistently higher rates of adverse events in women compared with men.^1^, ^2^, ^3^, ^4^, ^5^, ^6^, ^7^ The unsolved issue remains why?

### The Current Study

The main findings of the current study are: (1) women have lower procedural success as assessed by post‐PCI TIMI 3 flow regardless of time to hospital presentation; (2) time to hospital presentation correlates with 30‐day mortality; (3) for late STEMI presentations, women have higher mortality rates than men; (4) when women and men present for STEMI within 120 minutes of symptom onset, their outcomes are equally positive. These findings suggest the existence of an important sex‐by‐PCI treatment interaction. Furthermore, these findings demonstrate that delay to hospital presentation modifies the effects of PCI between sexes. Delay and suboptimal postprocedural TIMI flow grade are variables independently associated with excess mortality in women, suggesting complementary mechanisms of reduced survival. These outcomes raise several significant methodological and clinical issues.

### Women Have Lower Procedural Success

We know that failure to achieve post‐PCI TIMI 3 flow correlates with mortality.^38^, ^39^ The different twist of this study is that it demonstrates that failure to achieve post‐PCI TIMI 3 is one of the keys to explain mortality difference between sexes. The reasons for these procedure‐associated differences in TIMI flow rates cannot be explained by the data of this registry and should be further investigated. We can just assume that suboptimal reperfusion after primary PCI is related to microvascular disorders resulting from coronary vasospasm secondary to endothelial injury and/or distal embolization,^40^ and women have a higher frequency of coronary plaque erosion and microembolization that could result in more microvascular dysfunction than men.^41^ The central point we must underline, however, is that women with STEMI have a worse outcome, and this is likely attributable to a combination of factors. Indeed, only 2.5% of women and 0.5% of men died with a post‐PCI TIMI flow grade ≤2, whereas the overall mortality rates were much greater (ie, 5.9% and 2.3%, respectively). Thus, other mechanisms for excess mortality in women are operating, which cannot be explained by established cardiovascular risk factors or whether patients received conventional treatment by primary PCI.

### Time to Hospital Presentation Correlates With 30‐Day Mortality

Time to reperfusion is clearly linked with myocardial salvage, and it makes sense that more rapid reperfusion should impact outcome. Myocardial cell death starts soon after arterial occlusion, and the myocardial salvage one can expect from reperfusion decreases sharply between 2 and 3 hours.^42^ After 120 minutes, the benefit with respect to myocardial salvage is of limited magnitude. Previous studies have documented a strict relationship between total ischemic time and 1‐year mortality in patients treated with primary PCI^43^: every 30‐minute delay before reperfusion, the relative risk of mortality increases by 7.5%. North American and European guidelines recommend primary PCI as the preferred reperfusion strategy over thrombolysis in patients with STEMI, provided it can be performed within 120 minutes from diagnosis.^44^, ^45^ The American Heart Association established “Mission: Lifeline” to develop STEMI systems of care that would improve timely access to PCI.^46^ However, despite the increased focus on delays in STEMI care, it is difficult to translate guideline recommendations into clinical practice. The NCDR (National Cardiovascular Data Registry) found that only 15% of patients are treated within 120 minutes from symptom onset.^47^ In the current study, we examined the prehospital and hospital components of the total ischemic time: time from symptom onset to hospital presentation and door‐to‐balloon time. We found that the median time from symptom onset to hospital presentation ranged from 280 minutes in women to 240 minutes in men and that the median door‐to‐balloon times of women and men were 40 and 38 minutes, respectively. Much more important, we found that the proportions of men and women with time from symptom onset to hospital presentation within 120 minutes were only 23.2% and 29.1%, respectively. Women and men with the longer delay time had the higher risk of 30‐day mortality compared with those with shorter delay times. Given these data, we focused more on time from symptom onset to hospital presentation rather than focusing on door‐to‐balloon time. Indeed, the median door‐to‐balloon times of our patients were generally within the guideline‐endorsed time frame of ≤90 minutes. Door‐to‐balloon time only measures the delay to reperfusion once patients reach the hospitals, which constitutes a smaller fraction of the total ischemic time. Women were more likely to exceed reperfusion times than men in the prehospital phase of the total ischemic time.

### Early Versus Late STEMI Presentation and Sex Difference in Outcomes

The main concern was that sex is correlated with delays, and sex effect may have been masked by the delay effect. Patients were, therefore, stratified in 2 delay cohorts: those with time of symptom onset to hospital presentation up to 120 minutes or later and those with time within 120 minutes. The cutoff of 120 minutes was based on previous work.^4^, ^14^, ^27^, ^33^, ^48^, ^49^ The main finding of such analysis can be summarized as follows. For late STEMI presentations, women have higher mortality rates than men (OR, 2.03; 95% CI, 1.23–3.33). As the delay to hospital presentation decreased to <120 minutes, the cardiovascular risk of women became similar to that of men (OR, 1.28; 95% CI, 0.35–4.69). Interestingly, women have lower procedural success, as assessed by post‐PCI TIMI 3 flow, regardless of time to hospital presentation, which suggests that delay to hospital presentation and suboptimal postprocedural coronary blood flow are independent mechanisms responsible for sex differences in outcomes. As so, female sex may be a biological variable.^50^

### Female Sex as a Biological Variable

Little attention has been paid to provide experimental designs for comparative assessment of sex‐specific modifications in myocardial tissue and blood flow during myocardial infarction. Few explanations, therefore, may account for our findings. One possibility is that the initial process of infarct healing after STEMI is different among women and men. Female animals show a greater number of macrophages in their ischemic areas.^51^ Macrophages facilitate inward remodeling in arterial vessels, causing further reduction in blood flow with poorer myocardial perfusion. Another possibility is that impaired myocardial perfusion after PCI may be facilitated in women because they have smaller coronary artery size. A report from the Zwolle investigators^52^ demonstrated a significant association between small vessel size (<3 mm) and poor perfusion. As a final point, susceptibility to ischemia injury may also be sex dependent.^53^ Perhaps reduced collateral flow in women may account for more vulnerability to prolonged untreated ischemia after coronary obstruction and more reperfusion injury. This may lead to more complications, including a higher incidence of shock, ventricular septal rupture, and severe mitral regurgitation.

### Testing Hypotheses by IPTW Models

Estimating the effect of treatments on sex‐related responses is one of the crucial problems in resolving gender/sex issues. Indeed, treatment disparities may account for a portion of adverse outcomes, leaving uncertainty about the size of the problem and comparability of different series reported.^28^ Matched‐pairs designs are commonly implemented in the field of medical research to eliminate confounding and improve study efficiency. We matched patients using IPTW models. IPTW is based on the propensity score and has specific advantages in comparison with regression adjustment. Advantages include the ability to present a balance of covariates, which removes most of the confounding, a property that would be expected under randomization,^22^ and the ability to control for many covariates. Matching by IPTW reduces statistical power to a lower extent than adjustments in the model.^54^, ^55^

### Multiple Mediators of Excess Mortality in Women

We examined whether the combination of suboptimal post‐PCI blood flow and longer delays from symptom onset to hospital presentation may increase the risk of death in women compared with men, over and beyond the separate effect of each single factor. We defined 4 clinical scenarios. Patients admitted to hospital within 120 minutes having post‐PCI complete restoration of blood flow (scenario 1) were the most robust patients because they had the lowest mortality (Table 4). Patients admitted to hospital later and having suboptimal postprocedural flow (scenario 2) were the frailest subjects (Table S9). However, their outcomes did not differ so much from those of patients presenting within 120 minutes from their symptom onset, but still with post‐PCI TIMI flow grades 0 to 2 (scenario 3) (Table S8). Scenario 4 (Table 4) included patients presenting to hospital later than 120 minutes but with post‐PCI complete restoration of blood flow. In these patients, mortality rates were twice as high as those reported in scenario 1, but consistently less than those seen in patients with TIMI flow grades 0 to 2 (scenarios 2 and 3). These findings indicate that female sex is associated with multiple independent pathways leading to excess mortality after primary PCI for STEMI. It is remarkable that post‐PCI TIMI flow grade 0 to 2 is associated with a much higher risk of mortality compared with the risk for patients with normal post‐PCI TIMI flow grade. This relationship persists regardless of time to hospital presentation, and its magnitude is much greater for women than men. Suboptimal post‐PCI blood flow accounts for a part of the observed sex differences in outcomes. Further studies are required to clarify the mechanisms underlying the persisting female disadvantage in patients with longer delays to hospital presentation. This information is of foremost importance to reduce the sex gap in mortality.

### Myocardial Infarction in Younger Versus Older Women

Sex differences in mortality rates have been shown to be especially higher in the younger age group (<60 years).^5^, ^28^, ^29^, ^30^, ^56^ Accordingly, we studied sex differences in post‐PCI TIMI flow sorted by younger versus older age. Women had higher rates of post‐PCI TIMI flow grade 0 to 2 than men only in patients aged ≥60 years. This is consistent with the hypothesis that the association between female sex and excess mortality in STEMI is not attributable to only 1 factor (namely, post‐PCI TIMI flow). An important reason for the relatively higher mortality rates in younger women may be the occurrence of coronary spasm or dysfunction a few days after PCI.^57^ Diagnosis can be difficult, given the transience of coronary spasm, and might require more sophisticated diagnostic approaches than those used in current practice, as reflected by our registry study.

### Limitations

We cannot rule out that more men and women died before presentation to the hospitals; therefore, the mortality rates of the current study only reflect those of patients who reach the hospital. As an observational study, we cannot completely exclude residual confounding, even using IPTW analyses. On the other hands, one cannot randomize patients to sex. Because catheterization data were mandated as part of the registry protocol only in centers with PCI facilities, patient selection may have introduced potential referral bias. However, the external validity of this study is confirmed by the proportion of patients with post‐PCI TIMI flow grade 0 to 2 in the overall population, which is similar to previous estimates.^58^ Furthermore, the demographics, characteristics, and 30‐day mortality rates of patients in this study were similar to those reported by recent research.^59^ Finally, results may not be definitive without replication.

## Conclusions

To date, most studies focused only on female sex, whereas little or no specific data on comparison between sexes are available.^6^, ^60^, ^61^ The current study is the first investigation that estimated and reported heterogeneity of treatment effects after primary PCI among women and men. We provided data disaggregated by sex and gave insights into how sex may be relevant as a biological variable. Female sex has a distinct cardiovascular blood flow reactivity after primary PCI, regardless of time to hospital presentation. Furthermore, women are more vulnerable to prolonged untreated ischemia. For late STEMI presentations, women have a 2‐fold likelihood of mortality compared with men. These results provide motivation for adequate consideration of both sexes in experiments and disaggregation of data by sex, which may allow for sex‐based comparisons and may inform clinical interventions.

## Disclosures

None.

## Supporting information


**Data S1.** Supplemental methods.
**Table S1.** Characteristics of Patients Undergoing Primary PCI Within 12 Hours From Symptoms Onset and Excluded From the Analysis
**Table S2.** General Logistic Regression and Regression Coefficients in the Propensity Score Model in the Overall Study Population of Women Versus Men
**Table S3.** Angiographic and Procedural Characteristics
**Table S4.** TIMI Flow Grade ≤1: Inverse Probability of Treatment Weighting: Women Versus Men
**Table S5.** Multivariate Analysis of Factors Associated With Post PCI TIMI Flow Grade ≤2
**Table S6.** Multivariate Analysis of Factors Associated With 30‐Day Mortality
**Table S7.** Delay to Reperfusion for STEMI Sorted by Sex
**Table S8.** Inverse Probability of Treatment Weighting: Timely (<120‐Minutes) PCI Treatment Effect on 30‐Day Mortality in Patients With TIMI Flow Grade 0 to 2
**Table S9.** Inverse Probability of Treatment Weighting: Delayed (>120‐Minutes) Treatment Effects on 30‐Day Mortality in Patients With TIMI Flow Grade 0 to 2
**Table S10.** Inverse Probability of Treatment Weighting: Women Versus Men in Patients Aged 60 Years or Over
**Table S11.** Inverse Probability of Treatment Weighting: Women Versus Men in Patients Aged <60 Years
**Figure S1.** Thirty‐day mortality in early presenters (within 2 hours from symptom onset to admission) and late presenters (≥120 minutes) from symptoms onset to admission) in women versus men.
**Figure S2.** Delay to treatment and mortality in patients with TIMI flow grade 0 to 2.
