# Supplementary material for: Sex‐Specific Treatment Effects After Primary Percutaneous Intervention: A Study on Coronary Blood Flow and Delay to Hospital Presentation
Source: J Am Heart Assoc. 2019 Feb 15;8(4):e011190. doi: 10.1161/JAHA.118.011190 (PMC6405653; doi:10.1161/JAHA.118.011190)
Supplement: Supplementary file 1 — Data S1. Supplemental methods. Table S1. Characteristics of Patients Undergoing Primary PCI Within 12 Hours From Symptoms Onset and Excluded From the Analysis Table S2. General Logistic Regression and Regression Coefficients in the Propensity Score Model in the Overall Study Population of Women Versus Men Table S3. Angiographic and Procedural Characteristics Table S4. TIMI Flow Grade ≤1: Inverse Probability of Treatment Weighting: Women Versus Men Table S5. Multivariate Analysis of Factors Associated With Post PCI TIMI Flow Grade ≤2 Table S6. Multivariate Analysis of Factors Associated With 30‐Day Mortality Table S7. Delay to Reperfusion for STEMI Sorted by Sex Table S8. Inverse Probability of Treatment Weighting: Timely (<120‐Minutes) PCI Treatment Effect on 30‐Day Mortality in Patients With TIMI Flow Grade 0 to 2 Table S9. Inverse Probability of Treatment Weighting: Delayed (>120‐Minutes) Treatment Effects on 30‐Day Mortality in Patients With TIMI Flow Grade 0 to 2 Table S10. Inverse Probability of Treatment Weighting: Women Versus Men in Patients Aged 60 Years or Over Table S11. Inverse Probability of Treatment Weighting: Women Versus Men in Patients Aged <60 Years Figure S1. Thirty‐day mortality in early presenters (within 2 hours from symptom onset to admission) and late presenters (≥120 minutes) from symptoms onset to admission) in women versus men. Figure S2. Delay to treatment and mortality in patients with TIMI flow grade 0 to 2. [file JAH3-8-e011190-s001.pdf]

SUPPLEMENTAL MATERIAL

Data S1.

SUPPLEMENTAL METHODS

Inverse Probability of Treatment Weighting Analysis

We used Inverse Propensity of Treatment Weighting (IPTW) to balance the distribution of covariates between two patient groups. If *e* denotes the estimated propensity score (i.e. e=\hat{P}(Z=1 | x), where the patient x is included in patient group 1; then, 1-e = \hat{P}(Z=0 | x)), then the original sample is weighted by the following weights: Z/e+(1−Z)/ 1−e where Z represents the patient group. For instance, women (Z=1) are assigned a weight equal to the reciprocal of the propensity score (1/e), while men (Z=0) are assigned a weight equal to the reciprocal of one minus the propensity score (1/1-e). The weighting procedure for each sample balances the covariate distributions between two patient groups.

Nearest neighbor imputation algorithms

Nearest neighbor (NN) imputation algorithms are efficient methods to fill in missing data where each missing value on some records is replaced by a value obtained from related cases in the whole set of records. Thus, imputation for clinical features, whose missing rate exceeded 10%, was conducted using the average of measured values from k records (kNN) [1, 2]

NN algorithms are similarity-based methods that rely on distance metrics and results may change in relation to the similarity measure used to evaluate the distance between recipients and donors. In our work, we used the following norm as metric to evaluate distance:

(∑ni=1|xi−yi|p)1/p

Before imputation of the recipient Xi, the full set with no missing data C(X) was filtered to select a subset of features relevant to the missing variable to be imputed (Xi_miss). To this end, C(X) was

considered as a dataset in the context of a regression problem, where the variable with the missing data (Xmiss) was set as the class variable and the other q variables (X1, X2, …, Xq) as predictors. We also applied the RReliefF algorithm [3] The set was, therefore, filtered to select a subset Cs(X) ⊂ C(X) where (X1, X2, …, Xs) ⊂ (X1, X2, …, Xq) and s < q. In the present context, we set the number of neighbors for RReliefF equal to 10 and set s as 10 %, 20 % or 30 % of q. As C(X) is invariant to Xi, the filtering step was performed only once before the NN imputation step that, on the contrary was performed separately for each Xi.

Table S1. Characteristics of patients undergoing primary PCI within 12 hours from symptoms onset and excluded from the analysis.

Characteristics	Overall population N=2994	Women N=856	Men N=2138	P-value
Age, years	60.3 ± 11.3	63.9 ± 10.9	58.9 ± 11.2	<0.001
Median age, years (IQR)	60 (53 – 68)	64 (57 – 72)	59 (51 – 66)	<0.001
**Cardiovascular risk factors, n (%)**				
Family history of CAD	1115 (37.2)	330 (38.6)	785 (36.7)	0.350
Diabetes	680 (22.7)	269 (31.4)	411 (19.2)	<0.001
Hypertension	1952 (65.2)	635 (74.2)	1317 (61.6)	<0.001
Hypercholesterolemia	1195 (39.9)	338 (39.5)	857 (40.1)	0.763
Current smoking	1237 (41.3)	261 (30.5)	976 (45.7)	<0.001
Former smoking	59 (2.0)	8 (0.9)	51 (2.4)	0.002
**Previous cardiovascular disease, n (%)**				
Previous angina pectoris	279 (9.3)	104 (12.1)	175 (8.2)	0.002
Previous myocardial infarction	437 (14.6)	105 (12.3)	332 (15.5)	0.017
Previous PCI	649 (21.7)	190 (22.2)	459 (21.5)	0.664
Previous CABG	27 (0.9)	6 (0.7)	21 (1.0)	0.429
Peripheral artery disease	36 (1.2)	10 (1.2)	26 (1.2)	0.913
Previous heart failure	40 (1.3)	15 (1.8)	25 (1.2)	0.249
Previous stroke	118 (3.9)	48 (5.6)	70 (3.3)	0.008
**Clinical presentation, n (%)**				
ST-segment elevation in anterior leads	606 (20.2)	181 (21.1)	425 (19.9)	0.441
Killip Class ≥ 2	263 (8.8)	92 (10.7)	171 (8.0)	0.023
Systolic BP at baseline, mmHg	145.3 ± 19.1	145.6 ± 18.5	145.1 ± 19.4	0.551
Heart rate at baseline, beats/min	80.3 ± 13.0	80.5 ± 12.8	80.3 ± 13.0	0.709
Serum creatinine at baseline, µmol/liter	86.8 ± 47.9	86.0 ± 42.5	87.1 ± 49.7	0.630

Data are presented as number (%). CABG, coronary-artery bypass graft; CAD, coronary artery disease; IQR, interquartile range, PCI, percutaneous coronary intervention; SD, standard deviation

Table S2. General logistic regression and regression coefficients in the propensity score model in the overall study population of women versus men.

	β	SE	T statistics	p-value
**Constant term (α)**	-1.695	0.517	-3.281	0.0010
Age, decades	0.538	0.053	10.197	<0.0001
**Cardiovascular risk factors**				
Family history of CAD	0.390	0.108	3.610	0.0003
Diabetes	-0.010	0.119	-0.088	0.9299
Hypertension	0.530	0.115	4.599	<0.0001
Hypercholesterolemia	0.262	0.103	2.551	0.0107
Current smoking	-0.423	0.114	-3.701	0.0002
Former smoking	-1.211	0.182	-6.640	<0.0001
**Previous cardiovascular disease**				
Previous angina pectoris	0.165	0.125	1.326	0.1848
Previous myocardial infarction	0.281	0.237	-1.184	0.2365
Previous PCI	-0.570	0.375	-1.520	0.1285
Previous CABG	0.000	0.000	0.000	-
Peripheral artery disease	-0.332	0.524	-0.614	0.5392
Previous heart failure	-0.361	0.232	-1.555	0.1200
Previous stroke	-0.025	0.323	-0.077	0.9384
**Clinical presentation**				
ST-segment elevation in anterior leads	-0.315	0.107	-2.955	0.0031
Killip Class≥ 2	0.403	0.131	3.071	0.0021
Systolic blood pressure at baseline, mmHg	-0.007	0.002	-3.477	0.0005
Heart rate at baseline, beats/min	-0.001	0.003	-0.253	0.8002
Serum creatinine at baseline, µmol/liter	-0.022	0.002	-9.686	<0.0001

Optimized regression coefficient (ß) and constant term(α) for the logistic regression. CABG, coronary artery bypass graft; CAD, coronary artery disease; PCI, percutaneous coronary intervention

Table S3. Angiographic and Procedural Characteristics.

	Women (n=673)	Men (n=1923)	P-value
Multivessel disease, n (%)	305 (45.3)	857 (44.6)	0.735
Acute vessel closure, n (%)	3 (0.4)	5 (0.3)	0.510
Pre-procedural TIMI flow grade 3, n (%)	174 (25.9)	601 (31.3)	0.007
Bifurcation lesion, n (%)	11 (1.6)	40 (2.1)	0.448
Acute thrombosis, n (%)	5 (0.7)	10 (0.5)	0.547
Ischemic stroke, n (%)	3 (0.4)	4 (0.2)	0.391
Minor bleeding, n (%)	7 (1.0)	18 (0.9)	0.817
Major bleeding, n (%)	3 (0.4)	7 (0.4)	0.779

TIMI, thrombolysis In Myocardial Infarction

Table S4. TIMI flow grade ≤ 1: inverse probability of treatment weighting: women versus men.

Characteristics	Women (n=673)	Men (n=1923)	*P* value
Age, years	61.8 ± 11.6	60.3 ± 11.4	0.003
**Cardiovascular risk factors, %**			
Family history of CAD	34.9	31.9	0.153
Diabetes	22.5	21.2	0.480
Hypertension	68.4	65.0	0.109
Hypercholesterolemia	46.6	44.0	0.243
Current smoking	47.9	50.2	0.304
Former smoking	9.5	12.6	0.031
**Previous cardiovascular disease, %**			
Previous angina pectoris	20.7	19.4	0.466
Previous myocardial infarction	5.6	7.1	0.180
Previous PCI	1.9	3.9	0.013
Previous CABG	0	0.4	0.112
Peripheral artery disease	0.7	1.2	0.286
Previous heart failure	5.2	5.1	0.919
Previous stroke	3.0	2.4	0.398
**Clinical presentation at admission, %**			
ST-segment elevation in anterior leads	37.4	37.7	0.890
Killip Class ≥2	19.2	19.3	0.954
Systolic blood pressure at baseline, mmHg	134.8 ± 27.6	136.0 ± 24.3	0.324
Heart rate at baseline, beats/min	79.0 ± 17.2	79.2 ± 17.4	0.809
Serum creatinine at baseline, µmol/liter	77.8 ± 28.2	91.7 ± 46.4	<0.0001
**Outcomes**			
TIMI flow grade ≤ 1, %	5.8	3.9	0.0397
TIMI flow grade ≤ 1, OR (95% CI)	1.51 (1.02 – 2.26)		0.0411

Values are %, mean ± SD or odd ratio (95% confidence intervals)
CABG, denotes coronary-artery bypass graft; CAD, coronary artery disease; CI confidence interval; OR, odds radio; PCI, percutaneous coronary intervention; SD, standard deviation; TIMI, Thrombolysis In Myocardial Infarction

Table S5. Multivariate analysis of factors associated with post PCI TIMI flow grade ≤2.

**Characteristics**	**OR**	**95% CI**	**p-value**
Women	1.68	1.15 – 2.44	0.003
Age, decades	1.33	1.12 – 1.58	0.0006
**Cardiovascular risk factors**			
Family history of CAD	0.73	0.49 – 1.09	0.061
Diabetes	0.90	0.60 – 1.37	0.317
Hypertension	1.05	0.70 – 1.57	0.413
Hypercholesterolemia	0.97	0.68 – 1.39	0.441
Current smoking	1.34	0.88 – 2.05	0.083
Former smoking	1.48	0.87 – 2.54	0.076
**Previous cardiovascular disease**			
Previous angina pectoris	1.07	0.70 – 1.64	0.373
Previous myocardial infarction	0.82	0.36 – 1.86	0.320
Previous PCI	0.34	0.07 – 1.54	0.080
Previous CABG	0.00	0.00 – Inf	-
Peripheral artery disease	5.07	1.98- 12.99	0.0004
Previous heart failure	0.50	0.21 – 1.15	0.051
Previous stroke	0.81	0.29 – 2.31	0.348
**Clinical presentation**			
ST-segment elevation in anterior leads	1.88	1.32 – 2.69	0.0003
Killip Class ≥ 2	2.28	1.55 – 3.34	<0.001
Systolic blood pressure at baseline, mmHg	0.99	0.99 – 1.00	0.054
Heart rate at baseline, beats/min	1.01	1.00 – 1.01	0.0002
Serum creatinine at baseline, µmol/liter	1.00	1.00 – 1.01	0.0002

CABG, coronary-artery bypass graft; CAD, coronary artery disease; CI, confidence interval; OR, odds radio; PCI, percutaneous coronary intervention; TIMI, thrombolysis In Myocardial Infarction

Table S6. Multivariate analysis of factors associated with 30-day mortality.

Characteristics	OR	95% CI	p-value
Women	1.72	1.02 – 2.90	0.022
Age, decades	1.82	1.41 – 2.36	<0.001
**Cardiovascular risk factors**			
Family history of CAD	1.33	0.76 – 2.32	0.161
Diabetes	1.76	1.04 – 2.98	0.018
Hypertension	1.51	0.82 – 2.77	0.092
Hypercholesterolemia	0.51	0.29 – 0.88	0.008
Current smoking	1.17	0.64 – 2.14	0.306
Former smoking	0.47	0.17 – 1.31	0.073
**Previous cardiovascular disease**			
Previous angina pectoris	1.05	0.58 – 1.90	0.434
Previous myocardial infarction	0.91	0.35 – 2.37	0.424
Previous PCI	2.48	0.72 – 8.52	0.074
Previous CABG	0.00	0.00 - Inf	-
Peripheral artery disease	1.27	0.24 – 6.76	0.389
Previous heart failure	0.78	0.33 – 1.83	0.282
Previous stroke	2.67	0.95 – 7.45	0.031
**Clinical presentation**			
ST-segment elevation in anterior leads	0.89	0.53 – 1.51	0.335
Killip Class ≥ 2	5.19	3.03 – 8.88	<0.001
Systolic blood pressure at baseline, mmHg	0.97	0.96 – 0.98	<0.001
Heart rate at baseline, beats/min	1.01	1.00 – 1.02	0.021
Serum creatinine at baseline, µmol/liter	1.00	1.00 – 1.01	<0.001

CABG, coronary-artery bypass graft; CAD, coronary artery disease; CI, confidence intervals; OR, odds radio; PCI, percutaneous coronary intervention

Table S7. Delay to reperfusion for STEMI sorted by sex.

Characteristics	Women N=673	Men N=1923	P-value
Median time from symptoms onset to admission (IQR), min	280 (170 - 498)	240 (145 - 430)	0.383
Time from symptoms onset to admission<120 minutes, n (%)	156 (23.2)	560 (29.1)	0.002
Median time from door to balloon (IQR), min	40 (20 – 75)	38 (20 – 75)	0.677

Data are presented as median (IQR) or number (%)

IQR denotes interquartile range

Table S8. Inverse probability of treatment weighting: timely (<120-minutes) PCI treatment effect on 30-day mortality in in patients with TIMI flow grade 0-2.

Characteristics	Women (N=13)	Men (N=11)	p-value
Age, years	70.5 ± 16.3	63.3 ± 12.0	0.223
**Cardiovascular risk factors, %**			
Family history of CAD	38.5	18.2	0.287
Diabetes	7.7	27.3	0.240
Hypertension	69.2	72.7	0.858
Hypercholesterolemia	46.2	36.4	0.645
Current smoking	30.8	63.6	0.119
Former smoking	7.7	18.2	0.478
**Previous cardiovascular disease, %**			
Previous angina pectoris	30.8	18.2	0.493
Previous myocardial infarction	0	0	-
Previous PCI	7.7	0	0.337
Previous CABG	0	0	-
Peripheral artery disease	0	0	-
Previous heart failure	7.7	0	0.337
Previous stroke	7.7	0	0.337
**Clinical presentation**			
ST-segment elevation in anterior leads, %	61.5	90.9	0.094
Killip Class ≥ 2, %	53.8	9.1	0.016
Systolic blood pressure at baseline, mmHg	130.4 ± 16.1	139.0 ± 32.7	0.439
Heart rate at baseline, beats/min	87.3 ± 17.4	90.5 ± 21.9	0.696
Serum creatinine at baseline, µmol/liter	91.2 ± 52.3	134.9 ± 88.0	0.169
**Outcomes**			
Primary outcome: 30-day mortality, %	30.8	9.1	0.194
Odd ratio (95% CI)	4.19 (0.33 – 239.96)		0.327

Data are presented as percentages (%).

CABG, coronary-artery bypass graft; CAD coronary artery disease; CI confidence intervals; OR odds radio; PCI percutaneous coronary intervention; SD, standard deviation

Table S9. Inverse probability of treatment weighting: delayed (≥120-minutes) treatment effects on 30-day mortality in in patients with TIMI flow grade 0-2.

Characteristics	Women (N=48)	Men (N=85)	p-value
Age, years	69.1 ± 10.5	62.9 ± 10.9	0.001
**Cardiovascular risk factors, %**			
Family history of CAD	20.8	23.5	0.720
Diabetes	25.0	23.5	0.851
Hypertension	77.1	68.2	0.269
Hypercholesterolemia	45.8	44.7	0.901
Current smoking	25.0	50.6	0.003
Former smoking	8.3	18.8	0.076
**Previous cardiovascular disease, %**			
Previous angina pectoris	22.9	22.4	0.941
Previous myocardial infarction	4.2	7.1	0.475
Previous PCI	0	1.2	0.320
Previous CABG	0	0.4	-
Peripheral artery disease	4.2	7.1	0.475
Previous heart failure	6.2	3.5	0.505
Previous stroke	4.2	2.4	0.590
**Clinical presentation at admission**			
ST-segment elevation in anterior leads, %	47.9	52.9	0.582
Killip Class ≥ 2, %	58.3	35.3	0.011
Systolic blood pressure at baseline, mmHg	130.0 ± 31.3	132.3 ± 24.2	0.666
Heart rate at baseline, beats/min	85.9 ± 21.2	80.7 ± 18.7	0.161
Serum creatinine at baseline, µmol/liter	112.9 ± 112.1	115.6 ± 105.5	0.892
**Outcomes**			
Primary outcome: 30-day mortality, %	27.1	12.9	0.061
Odds Ratio (95% CI)	2.48 (0.92 – 6.80)		0.059

Data are presented as percentages (%).CABG, coronary-artery bypass grafting; CAD coronary artery disease; CI confidence intervals; OR odds radio; PCI percutaneous coronary intervention; SD, standard deviation

Table S10. Inverse probability of treatment weighting: women versus men in patients aged 60 year or over.

Characteristics	Women (n=462)	Men (n=878)	*P* value
Age, years	69.4 ± 7.5	69.4 ± 7.4	0.908
**Cardiovascular risk factors, %**			
Family history of CAD	22.4	22.1	0.900
Diabetes	25.3	25.9	0.811
Hypertension	76.6	74.4	0.376
Hypercholesterolemia	43.3	43.2	0.972
Current smoking	31.7	33.0	0.553
Former smoking	12.3	14.0	0.385
**Previous cardiovascular disease, %**			
Previous angina pectoris	21.9	22.7	0.739
Previous myocardial infarction	5.0	7.6	0.071
Previous PCI	2.0	3.9	0.062
Previous CABG	0	0.8	0.047
Peripheral artery disease	1.8	1.6	0.785
Previous heart failure	5.9	6.7	0.571
Previous stroke	3.7	3.3	0.702
**Clinical presentation at admission, %**			
ST-segment elevation in anterior leads	38.7	37.2	0.591
Killip Class ≥2	17.0	16.7	0.889
Systolic blood pressure at baseline, mmHg	136.4 ± 29.2	136.5 ± 25.3	0.936
Heart rate at baseline, beats/min	79.5 ± 18.0	79.4 ± 19.2	0.901
Serum creatinine at baseline, µmol/liter	84.5 ± 25.6	95.2 ± 48.5	<0.0001
**Outcomes**			
TIMI flow grade ≤ 2, %	12.1	8.1	0.0179
TIMI flow grade ≤2, OR (95% CI)	1.56 (1.08 – 2.26)		0.0188

Values are %, mean ± SD or odd ratio (95% confidence intervals)

CABG, denotes coronary-artery bypass graft; CAD, coronary artery disease; CI confidence interval; OR, odds radio; PCI, percutaneous coronary intervention; SD, standard deviation; TIMI, Thrombolysis In Myocardial Infarction

Table S11. Inverse probability of treatment weighting: women versus men in patients aged < 60 year.

Characteristics	Women (n=211)	Men (n=1045)	*P* value
Age, years	51.1 ± 6.1	50.9 ± 6.4	0.563
**Cardiovascular risk factors, %**			
Family history of CAD	45.3	40.4	0.187
Diabetes	18.0	16.5	0.595
Hypertension	53.2	55.6	0.523
Hypercholesterolemia	45.2	43.9	0.729
Current smoking	70.5	67.1	0.336
Former smoking	7.3	11.3	0.085
**Previous cardiovascular disease, %**			
Previous angina pectoris	15.3	16.1	0.773
Previous myocardial infarction	6.4	6.4	1.000
Previous PCI	3.0	3.8	0.574
Previous CABG	0	0.1	0.608
Peripheral artery disease	0	1.0	0.137
Previous heart failure	3.9	3.4	0.719
Previous stroke	2.1	1.5	0.524
**Clinical presentation at admission, %**			
ST-segment elevation in anterior leads	36.3	38.0	0.642
Killip Class ≥2	7.6	7.8	0.921
Systolic blood pressure at baseline, mmHg	134.7 ± 24.2	135.3 ± 23.5	0.741
Heart rate at baseline, beats/min	78.3 ± 15.2	79.3 ± 16.1	0.397
Serum creatinine at baseline, µmol/liter	67.0 ± 14.2	86.9 ± 42.8	<0.0001
**Outcomes**			
TIMI flow grade ≤ 2, %	2.1	3.7	0.2459
TIMI flow grade ≤2, OR (95% CI)	0.56 (0.21 – 1.51)		0.2522

Values are %, mean ± SD or odd ratio (95% confidence intervals)

CABG, denotes coronary-artery bypass graft; CAD, coronary artery disease; CI confidence interval; OR, odds radio; PCI, percutaneous coronary intervention; SD, standard deviation; TIMI, Thrombolysis In Myocardial Infarction

Figure S1. 30-day mortality in early presenters (within 2 hours from symptom onset to admission) and late presenters (≥120 minutes) from symptoms onset to admission) in women versus men.

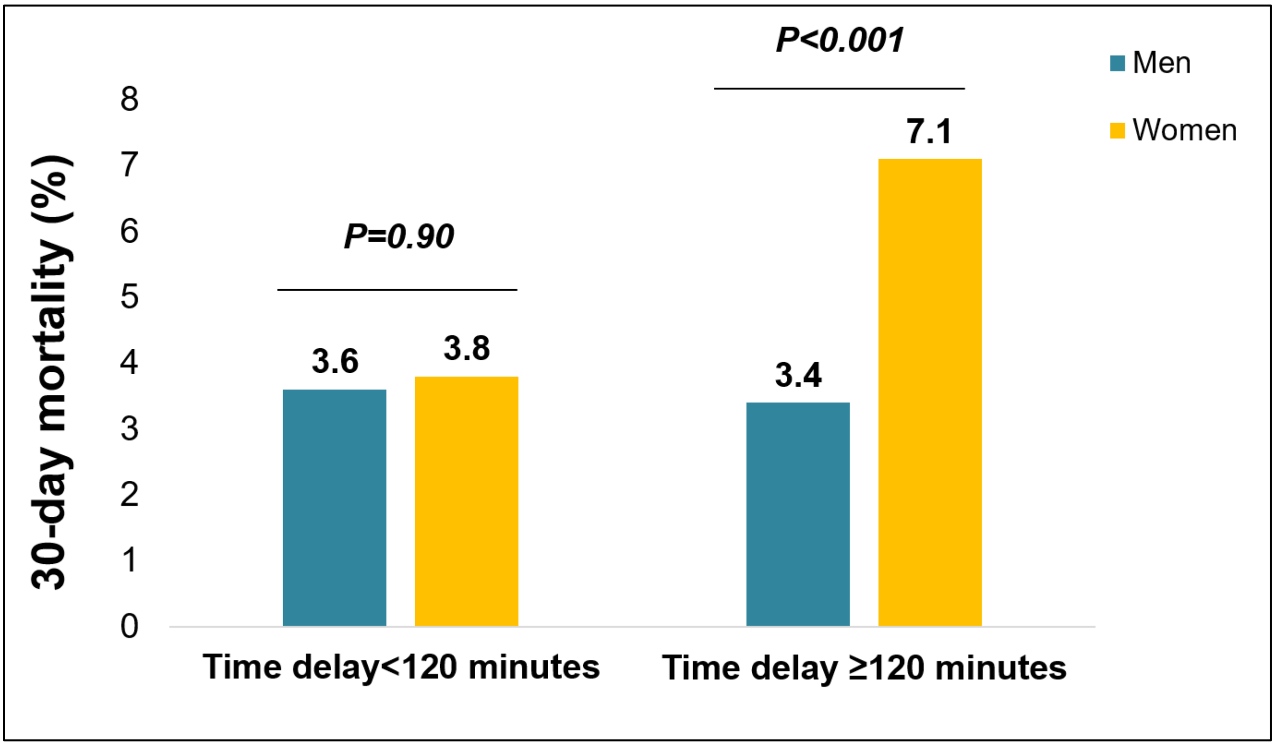

Figure S2. Delay to treatment and mortality in patients with TIMI flow grade 0-2.

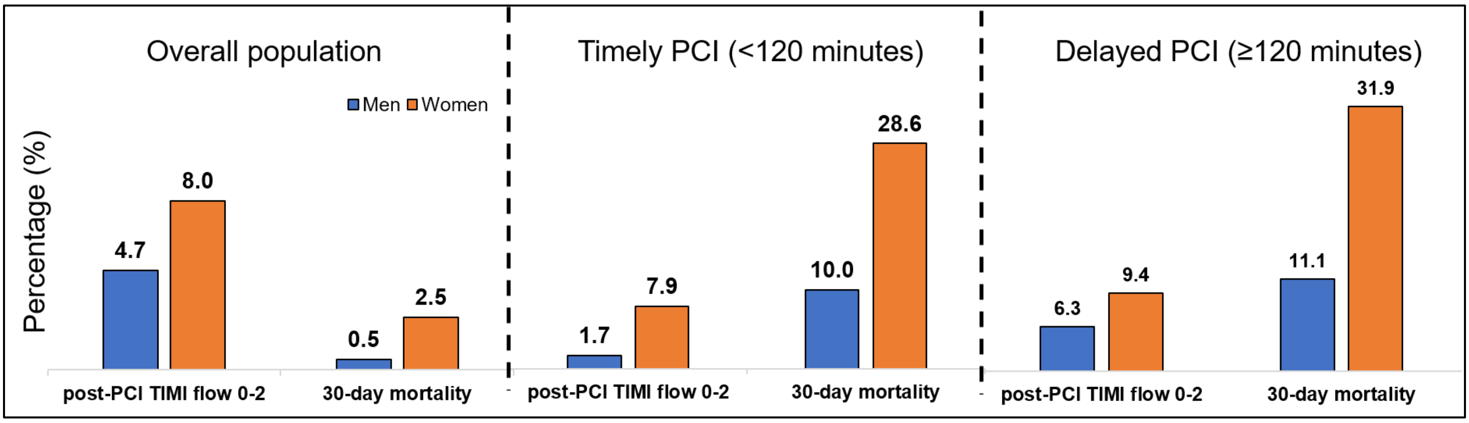

Supplemental References:

1. Troyanskaya O, Cantor M, Sherlock G, Brown P, Hastie T, Tibshirani R, Botstein D, Altman RB. Missing value estimation methods for DNA microarrays. *Bioinformatics.* 2001;17:520-5.

2. Beretta L, Santaniello A. Nearest neighbor imputation algorithms: a critical evaluation. *BMC Med Inform Decis Mak.* 2016;16 Suppl 3:74.

3. Kononenko I, Simec E, Robnik-Sikonja M. Overcoming the myopia of inductive learning algorithms with RELIEFF. *Appl Intell*. 1997;7:39-55.
