# Supplementary material for: Sex‐Specific Treatment Effects After Primary Percutaneous Intervention: A Study on Coronary Blood Flow and Delay to Hospital Presentation
Source: J Am Heart Assoc. 2019 Feb 15;8(4):e011190. doi: 10.1161/JAHA.118.011190 (PMC6405653; doi:10.1161/JAHA.118.011190)
Supplement: Supplementary file 2 [file JAH3-8-e011190-s002.docx]

**SUPPLEMENTAL MATERIAL**

**Sex Specific Treatment Effects after Primary Percutaneous Intervention.**

**A study on coronary blood flow and delay to hospital presentation**

TABLE OF CONTENTS

[SUPPLEMENTAL METHODS 2](#_Toc532401993)

[Inverse Probability of Treatment Weighting Analysis 2](#_Toc532401994)

[Nearest neighbour imputation algorithms 2](#_Toc532401995)

[Supplemental Figure 1. 30-day mortality in early presenters (within 2 hours from symptom onset to admission) and late presenters (≥120 minutes) from symptoms onset to admission) in women versus men. 4](#_Toc532401996)

[Supplemental Figure 2. Delay to treatment and mortality in patients with TIMI flow grade 0-2 5](#_Toc532401997)

[Supplemental Table 1. Characteristics of patients undergoing primary PCI within 12 hours from symptoms onset and excluded from the analysis. 6](#_Toc532401998)

[Supplemental Table 2. General logistic regression and regression coefficients in the propensity score model in the overall study population of women versus men 7](#_Toc532401999)

[Supplemental Table 3. Angiographic and Procedural Characteristics. 8](#_Toc532402000)

[Supplemental Table 4. TIMI flow grade ≤ 1: inverse probability of treatment weighting: women versus men. 9](#_Toc532402001)

[Supplemental Table 5. Multivariate analysis of factors associated with post PCI TIMI flow grade ≤2 10](#_Toc532402002)

[Supplemental Table 6. Multivariate analysis of factors associated with 30-day mortality 11](#_Toc532402003)

[Supplemental Table 7. Delay to reperfusion for STEMI sorted by sex. 12](#_Toc532402004)

[Supplemental Table 8.. Inverse probability of treatment weighting: timely (<120‐minutes) PCI treatment effect on 30-day mortality in in patients with TIMI flow grade 0-2 13](#_Toc532402005)

[Supplemental Table 9.. Inverse probability of treatment weighting: delayed (>120‐minutes) treatment effects on 30-day mortality in in patients with TIMI flow grade 0-2 14](#_Toc532402006)

[Supplemental Table 10. Inverse probability of treatment weighting: women versus men in patients aged 60 year or over. 15](#_Toc532402007)

[Supplemental Table 11. Inverse probability of treatment weighting: women versus men in patients aged < 60 year. 16](#_Toc532402008)

[REFERENCES: 17](#_Toc532402009)

# SUPPLEMENTAL METHODS

## Inverse Probability of Treatment Weighting Analysis

We used Inverse Propensity of Treatment Weighting (IPTW) to balance the distribution of covariates between two patient groups. If e denotes the estimated propensity score (i.e. e=\hat{P}(Z=1 | x), where the patient x is included in patient group 1; then, 1-e = \hat{P}(Z=0 | x)), then the original sample is weighted by the following weights: Z/e+(1−Z)/ 1−e where Z represents the patient group. For instance, women (Z=1) are assigned a weight equal to the reciprocal of the propensity score (1/e), while men (Z=0) are assigned a weight equal to the reciprocal of one minus the propensity score (1/1-e). The weighting procedure for each sample balances the covariate distributions between two patient groups.

## Nearest neighbour imputation algorithms

Nearest neighbour (NN) imputation algorithms are efficient methods to fill in missing data where each missing value on some records is replaced by a value obtained from related cases in the whole set of records. Thus, imputation for clinical features, whose missing rate exceeded 10%, was conducted using the average of measured values from k records (kNN) [1, 2]

NN algorithms are similarity-based methods that rely on distance metrics and results may change in relation to the similarity measure used to evaluate the distance between recipients and donors. In our work, we used the following norm as metric to evaluate distance:

(∑ni=1|xi−yi|p)1/p

Before imputation of the recipient Xi, the full set with no missing data C(X) was filtered to select a subset of features relevant to the missing variable to be imputed (Xi_miss). To this end, C(X) was considered as a dataset in the context of a regression problem, where the variable with the missing data (Xmiss) was set as the class variable and the other q variables (X1, X2, …, Xq) as predictors. We also applied the RReliefF algorithm [3] The set was, therefore, filtered to select a subset Cs(X) ⊂ C(X) where (X1, X2, …, Xs) ⊂ (X1, X2, …, Xq) and s < q. In the present context, we set the number of neighbours for RReliefF equal to 10 and set s as 10 %, 20 % or 30 % of q. As C(X) is invariant to Xi, the filtering step was performed only once before the NN imputation step that, on the contrary was performed separately for each Xi.

## **Supplemental Figure 1**. 30-day mortality in early presenters (within 2 hours from symptom onset to admission) and late presenters (≥120 minutes) from symptoms onset to admission) in women versus men.


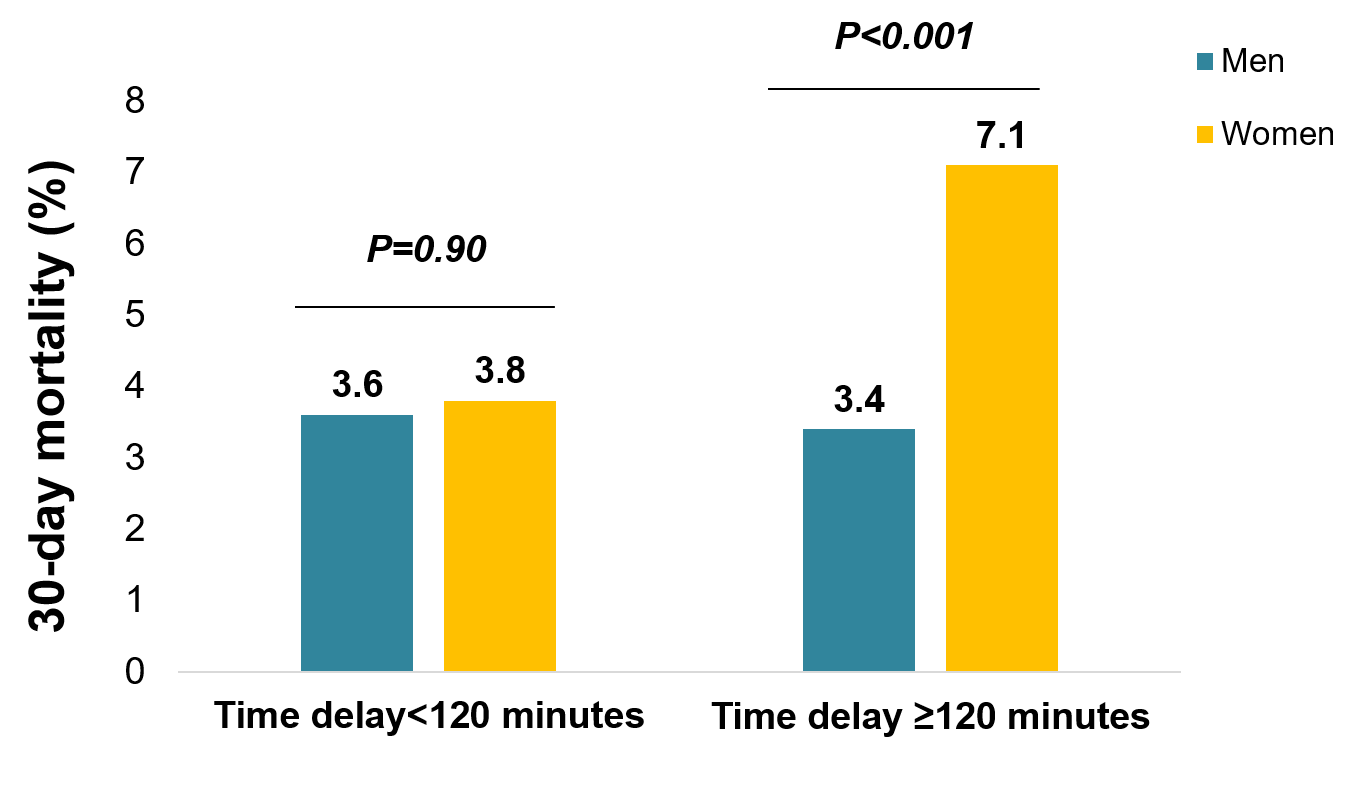


## **Supplemental Figure 2.** Delay to treatment and mortality in patients with TIMI flow grade 0-2


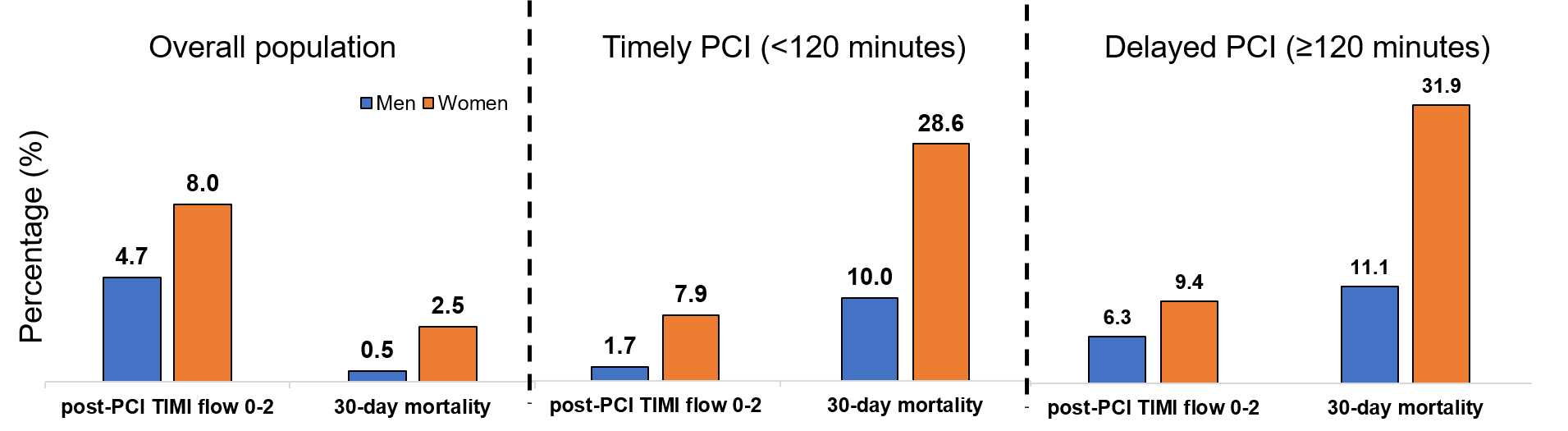


| Supplemental Table 1. Characteristics of patients undergoing primary PCI within 12 hours from symptoms onset and excluded from the analysis. | | | | |
| --- | --- | --- | --- | --- |
| **Characteristics** | **Overall population**  **N=2994** | **Women**  **N=856** | **Men**  **N=2138** | **P-value** |
| Age, years | 60.3 ± 11.3 | 63.9 ± 10.9 | 58.9 ± 11.2 | <0.001 |
| Median age, years (IQR) | 60 (53 – 68) | 64 (57 – 72) | 59 (51 – 66) | <0.001 |
| **Cardiovascular risk factors, n (%)** |  |  |  |  |
| Family history of CAD | 1115 (37.2) | 330 (38.6) | 785 (36.7) | 0.350 |
| Diabetes | 680 (22.7) | 269 (31.4) | 411 (19.2) | <0.001 |
| Hypertension | 1952 (65.2) | 635 (74.2) | 1317 (61.6) | <0.001 |
| Hypercholesterolemia | 1195 (39.9) | 338 (39.5) | 857 (40.1) | 0.763 |
| Current smoking | 1237 (41.3) | 261 (30.5) | 976 (45.7) | <0.001 |
| Former smoking | 59 (2.0) | 8 (0.9) | 51 (2.4) | 0.002 |
| **Previous cardiovascular disease, n (%)** |  |  |  |  |
| Previous angina pectoris | 279 (9.3) | 104 (12.1) | 175 (8.2) | 0.002 |
| Previous myocardial infarction | 437 (14.6) | 105 (12.3) | 332 (15.5) | 0.017 |
| Previous PCI | 649 (21.7) | 190 (22.2) | 459 (21.5) | 0.664 |
| Previous CABG | 27 (0.9) | 6 (0.7) | 21 (1.0) | 0.429 |
| Peripheral artery disease | 36 (1.2) | 10 (1.2) | 26 (1.2) | 0.913 |
| Previous heart failure | 40 (1.3) | 15 (1.8) | 25 (1.2) | 0.249 |
| Previous stroke | 118 (3.9) | 48 (5.6) | 70 (3.3) | 0.008 |
| **Clinical presentation, n (%)** |  |  |  |  |
| ST-segment elevation in anterior leads | 606 (20.2) | 181 (21.1) | 425 (19.9) | 0.441 |
| Killip Class > 2 | 263 (8.8) | 92 (10.7) | 171 (8.0) | 0.023 |
| Systolic BP at baseline, mmHg | 145.3 ± 19.1 | 145.6 ± 18.5 | 145.1 ± 19.4 | 0.551 |
| Heart rate at baseline, beats/min | 80.3 ± 13.0 | 80.5 ± 12.8 | 80.3 ± 13.0 | 0.709 |
| Serum creatinine at baseline, µmol/liter | 86.8 ± 47.9 | 86.0 ± 42.5 | 87.1 ± 49.7 | 0.630 |
| Data are presented as number (%). Abbreviations: CABG, coronary-artery bypass graft; CAD, coronary artery disease; IQR, interquartile range, PCI, percutaneous coronary intervention; SD, standard deviation | | | | |

| Supplemental Table 2. General logistic regression and regression coefficients in the propensity score model in the overall study population of women versus men | | | | |
| --- | --- | --- | --- | --- |
|  | $\boldsymbol{\beta}$ | **SE** | **T statistics** | **p-value** |
| **Constant term (α)** | -1.695 | 0.517 | -3.281 | 0.0010 |
| Age, decades | 0.538 | 0.053 | 10.197 | <0.0001 |
| **Cardiovascular risk factors** |  |  |  |  |
| Family history of CAD | 0.390 | 0.108 | 3.610 | 0.0003 |
| Diabetes | -0.010 | 0.119 | -0.088 | 0.9299 |
| Hypertension | 0.530 | 0.115 | 4.599 | <0.0001 |
| Hypercholesterolemia | 0.262 | 0.103 | 2.551 | 0.0107 |
| Current smoking | -0.423 | 0.114 | -3.701 | 0.0002 |
| Former smoking | -1.211 | 0.182 | -6.640 | <0.0001 |
| **Previous cardiovascular disease** |  |  |  |  |
| Previous angina pectoris | 0.165 | 0.125 | 1.326 | 0.1848 |
| Previous myocardial infarction | 0.281 | 0.237 | -1.184 | 0.2365 |
| Previous PCI | -0.570 | 0.375 | -1.520 | 0.1285 |
| Previous CABG | 0.000 | 0.000 | 0.000 | - |
| Peripheral artery disease | -0.332 | 0.524 | -0.614 | 0.5392 |
| Previous heart failure | -0.361 | 0.232 | -1.555 | 0.1200 |
| Previous stroke | -0.025 | 0.323 | -0.077 | 0.9384 |
| **Clinical presentation** |  |  |  |  |
| ST-segment elevation in anterior leads | -0.315 | 0.107 | -2.955 | 0.0031 |
| Killip Class> 2 | 0.403 | 0.131 | 3.071 | 0.0021 |
| Systolic blood pressure at baseline, mmHg | -0.007 | 0.002 | -3.477 | 0.0005 |
| Heart rate at baseline, beats/min | -0.001 | 0.003 | -0.253 | 0.8002 |
| Serum creatinine at baseline, µmol/liter | -0.022 | 0.002 | -9.686 | <0.0001 |
| Optimized regression coefficient (ß) and constant term(α) for the logistic regression.  Abbreviations: CABG, coronary artery bypass graft; CAD, coronary artery disease; PCI, percutaneous coronary intervention | | | | |

| Supplemental Table 3. Angiographic and Procedural Characteristics. | | | |
| --- | --- | --- | --- |
|  | **Women**  **(n=673)** | **Men**  **(n=1923)** | **P-value** |
| Multivessel disease, n (%) | 305 (45.3) | 857 (44.6) | 0.735 |
| Acute vessel closure, n (%) | 3 (0.4) | 5 (0.3) | 0.510 |
| Pre-procedural TIMI flow grade 3, n (%) | 174 (25.9) | 601 (31.3) | 0.007 |
| Bifurcation lesion, n (%) | 11 (1.6) | 40 (2.1) | 0.448 |
| Acute thrombosis, n (%) | 5 (0.7) | 10 (0.5) | 0.547 |
| Ischemic stroke, n (%) | 3 (0.4) | 4 (0.2) | 0.391 |
| Minor bleeding, n (%) | 7 (1.0) | 18 (0.9) | 0.817 |
| Major bleeding, n (%) | 3 (0.4) | 7 (0.4) | 0.779 |
| Abbreviations: TIMI, thrombolysis In Myocardial Infarction | | | |

| Supplemental Table 4. TIMI flow grade ≤ 1: inverse probability of treatment weighting: women versus men. | | | | | | | | | | | |
| --- | --- | --- | --- | --- | --- | --- | --- | --- | --- | --- | --- |
| **Characteristics** | | **Women**  **(n=673)** | | | | **Men**  **(n=1923)** | ***P* value** | | | |  |
| Age, years | | 61.8 ± 11.6 | | | | 60.3 ± 11.4 | 0.003 | | | |  |
| **Cardiovascular risk factors, %** | |  | | | |  |  | | | |  |
| Family history of CAD | | 34.9 | | | | 31.9 | 0.153 | | | |  |
| Diabetes | | 22.5 | | | | 21.2 | 0.480 | | | |  |
| Hypertension | | 68.4 | | | | 65.0 | 0.109 | | | |  |
| Hypercholesterolemia | | 46.6 | | | | 44.0 | 0.243 | | | |  |
| Current smoking | | 47.9 | | | | 50.2 | 0.304 | | | |  |
| Former smoking | | 9.5 | | | | 12.6 | 0.031 | | | |  |
| **Previous cardiovascular disease, %** | |  | | | |  |  | | | |  |
| Previous angina pectoris | | 20.7 | | | | 19.4 | 0.466 | | | |  |
| Previous myocardial infarction | | 5.6 | | | | 7.1 | 0.180 | | | |  |
| Previous PCI | | 1.9 | | | | 3.9 | 0.013 | | | |  |
| Previous CABG | | 0 | | | | 0.4 | 0.112 | | | |  |
| Peripheral artery disease | | 0.7 | | | | 1.2 | 0.286 | | | |  |
| Previous heart failure | | 5.2 | | | | 5.1 | 0.919 | | | |  |
| Previous stroke | | 3.0 | | | | 2.4 | 0.398 | | | |  |
| **Clinical presentation at admission, %** | |  | | | |  | |  | | | |
| ST-segment elevation in anterior leads | | 37.4 | | | | 37.7 | 0.890 | | | |  |
| Killip Class ≥2 | | 19.2 | | | | 19.3 | 0.954 | | | |  |
| Systolic blood pressure at baseline, mmHg | | 134.8 ± 27.6 | | | | 136.0 ± 24.3 | 0.324 | | | |  |
| Heart rate at baseline, beats/min | | 79.0 ± 17.2 | | | | 79.2 ± 17.4 | 0.809 | | | |  |
| Serum creatinine at baseline, µmol/liter | | 77.8 ± 28.2 | | | | 91.7 ± 46.4 | <0.0001 | | | |  |
| **Outcomes** | |  | | | |  | |  | | | |
| TIMI flow grade ≤ 1, % | | 5.8 | | | 3.9 | | | 0.0397 | | | |
| TIMI flow grade ≤ 1, OR (95% CI) | | 1.51 (1.02 – 2.26) | | | | | | 0.0411 | | | |
| Values are %, mean ± SD or odd ratio (95% confidence intervals)  Abbreviations: CABG, denotes coronary-artery bypass graft; CAD, coronary artery disease; CI confidence interval; OR, odds radio; PCI, percutaneous coronary intervention; SD, standard deviation; TIMI, Thrombolysis In Myocardial Infarction | | | | | | | | | | | |
| Supplemental Table 5. Multivariate analysis of factors associated with post PCI TIMI flow grade ≤2 | | | | | | | | | | | |
| **Characteristics** | **OR** | | **95% CI** | | | | | | **p-value** | | |
| Women | 1.68 | | 1.15 – 2.44 | | | | | | 0.003 | | |
| Age, decades | 1.33 | | 1.12 – 1.58 | | | | | | 0.0006 | | |
| **Cardiovascular risk factors** |  | |  | | | | | |  | | |
| Family history of CAD | 0.73 | | 0.49 – 1.09 | | | | | | 0.061 | | |
| Diabetes | 0.90 | | 0.60 – 1.37 | | | | | | 0.317 | | |
| Hypertension | 1.05 | | 0.70 – 1.57 | | | | | | 0.413 | | |
| Hypercholesterolemia | 0.97 | | 0.68 – 1.39 | | | | | | 0.441 | | |
| Current smoking | 1.34 | | 0.88 – 2.05 | | | | | | 0.083 | | |
| Former smoking | 1.48 | | 0.87 – 2.54 | | | | | | 0.076 | | |
| **Previous cardiovascular disease** |  | |  | | | | | |  | | |
| Previous angina pectoris | 1.07 | | 0.70 – 1.64 | | | | | | 0.373 | | |
| Previous myocardial infarction | 0.82 | | 0.36 – 1.86 | | | | | | 0.320 | | |
| Previous PCI | 0.34 | | 0.07 – 1.54 | | | | | | 0.080 | | |
| Previous CABG | 0.00 | | 0.00 – Inf | | | | | | - | | |
| Peripheral artery disease | 5.07 | | 1.98- 12.99 | | | | | | 0.0004 | | |
| Previous heart failure | 0.50 | | 0.21 – 1.15 | | | | | | 0.051 | | |
| Previous stroke | 0.81 | | 0.29 – 2.31 | | | | | | 0.348 | | |
| **Clinical presentation** |  | | |  | | | | | |  | |
| ST-segment elevation in anterior leads | 1.88 | | 1.32 – 2.69 | | | | | | 0.0003 | | |
| Killip Class ≥ 2 | 2.28 | | 1.55 – 3.34 | | | | | | <0.001 | | |
| Systolic blood pressure at baseline, mmHg | 0.99 | | 0.99 – 1.00 | | | | | | 0.054 | | |
| Heart rate at baseline, beats/min | 1.01 | | 1.00 – 1.01 | | | | | | 0.0002 | | |
| Serum creatinine at baseline, µmol/liter | 1.00 | | 1.00 – 1.01 | | | | | | 0.0002 | | |
| Abbreviations: CABG, coronary-artery bypass graft; CAD, coronary artery disease; CI, confidence interval; OR, odds radio; PCI, percutaneous coronary intervention; TIMI, thrombolysis In Myocardial Infarction | | | | | | | | | | | |

| Supplemental Table 6. Multivariate analysis of factors associated with 30-day mortality | | | | | |
| --- | --- | --- | --- | --- | --- |
| **Characteristics** | **OR** | **95% CI** | | **p-value** | |
| Women | 1.72 | 1.02 – 2.90 | | 0.022 | |
| Age, decades | 1.82 | 1.41 – 2.36 | | <0.001 | |
| **Cardiovascular risk factors** |  |  | |  | |
| Family history of CAD | 1.33 | 0.76 – 2.32 | | 0.161 | |
| Diabetes | 1.76 | 1.04 – 2.98 | | 0.018 | |
| Hypertension | 1.51 | 0.82 – 2.77 | | 0.092 | |
| Hypercholesterolemia | 0.51 | 0.29 – 0.88 | | 0.008 | |
| Current smoking | 1.17 | 0.64 – 2.14 | | 0.306 | |
| Former smoking | 0.47 | 0.17 – 1.31 | | 0.073 | |
| **Previous cardiovascular disease** |  |  | |  | |
| Previous angina pectoris | 1.05 | 0.58 – 1.90 | | 0.434 | |
| Previous myocardial infarction | 0.91 | 0.35 – 2.37 | | 0.424 | |
| Previous PCI | 2.48 | 0.72 – 8.52 | | 0.074 | |
| Previous CABG | 0.00 | 0.00 - Inf | | - | |
| Peripheral artery disease | 1.27 | 0.24 – 6.76 | | 0.389 | |
| Previous heart failure | 0.78 | 0.33 – 1.83 | | 0.282 | |
| Previous stroke | 2.67 | 0.95 – 7.45 | | 0.031 | |
| **Clinical presentation** |  | |  | |  |
| ST-segment elevation in anterior leads | 0.89 | 0.53 – 1.51 | | 0.335 | |
| Killip Class ≥ 2 | 5.19 | 3.03 – 8.88 | | <0.001 | |
| Systolic blood pressure at baseline, mmHg | 0.97 | 0.96 – 0.98 | | <0.001 | |
| Heart rate at baseline, beats/min | 1.01 | 1.00 – 1.02 | | 0.021 | |
| Serum creatinine at baseline, µmol/liter | 1.00 | 1.00 – 1.01 | | <0.001 | |
|  |  |  | |  | |
| Abbreviations: CABG, coronary-artery bypass graft; CAD, coronary artery disease; CI, confidence intervals; OR, odds radio; PCI, percutaneous coronary intervention | | | | | |

| Supplemental Table 7. Delay to reperfusion for STEMI sorted by sex. | | | |
| --- | --- | --- | --- |
| **Characteristics** | **Women**  **N=673** | **Men**  **N=1923** | **P-value** |
| Median time from symptoms onset to admission (IQR), min | 280 (170 - 498) | 240 (145 - 430) | 0.383 |
| Time from symptoms onset to admission<120 minutes, n (%) | 156 (23.2) | 560 (29.1) | 0.002 |
| Median time from door to balloon (IQR), min | 40 (20 – 75) | 38 (20 – 75) | 0.677 |
| Data are presented as median (IQR) or number (%)  Abbreviations: IQR denotes interquartile range | | | |

| Supplemental Table 8.. Inverse probability of treatment weighting: timely (<120‐minutes) PCI treatment effect on 30-day mortality in in patients with TIMI flow grade 0-2 | | | | | | | | |
| --- | --- | --- | --- | --- | --- | --- | --- | --- |
| **Characteristics** | | **Women**  **(N=13)** | | **Men**  **(N=11)** | | **p-value** | | |
| Age, years | | 70.5 ± 16.3 | | 63.3 ± 12.0 | | 0.223 | | |
| **Cardiovascular risk factors, %** | |  | |  | |  | | |
| Family history of CAD | | 38.5 | | 18.2 | | 0.287 | | |
| Diabetes | | 7.7 | | 27.3 | | 0.240 | | |
| Hypertension | | 69.2 | | 72.7 | | 0.858 | | |
| Hypercholesterolemia | | 46.2 | | 36.4 | | 0.645 | | |
| Current smoking | | 30.8 | | 63.6 | | 0.119 | | |
| Former smoking | | 7.7 | | 18.2 | | 0.478 | | |
| **Previous cardiovascular disease, %** | |  | |  | |  | | |
| Previous angina pectoris | | 30.8 | | 18.2 | | 0.493 | | |
| Previous myocardial infarction | | 0 | | 0 | | - | | |
| Previous PCI | | 7.7 | | 0 | | 0.337 | | |
| Previous CABG | | 0 | | 0 | | - | | |
| Peripheral artery disease | | 0 | | 0 | | - | | |
| Previous heart failure | | 7.7 | | 0 | | 0.337 | | |
| Previous stroke | | 7.7 | | 0 | | 0.337 | | |
| **Clinical presentation** | |  | |  | |  | | |
| ST-segment elevation in anterior leads, % | | 61.5 | | 90.9 | | 0.094 | | |
| Killip Class ≥ 2, % | | 53.8 | | 9.1 | | 0.016 | | |
| Systolic blood pressure at baseline, mmHg | | 130.4 ± 16.1 | | 139.0 ± 32.7 | | 0.439 | | |
| Heart rate at baseline, beats/min | | 87.3 ± 17.4 | | 90.5 ± 21.9 | | 0.696 | | |
| Serum creatinine at baseline, µmol/liter | | 91.2 ± 52.3 | | 134.9 ± 88.0 | | 0.169 | | |
| **Outcomes** | |  | |  | |  | | |
| Primary outcome: 30-day mortality, % | | 30.8 | | 9.1 | | 0.194 | | |
| Odd ratio (95% CI) | | 4.19 (0.33 – 239.96) | | | | 0.327 | | |
| Data are presented as percentages (%).  Abbreviations: CABG, coronary-artery bypass graft; CAD coronary artery disease; CI confidence intervals; OR odds radio; PCI percutaneous coronary intervention; SD, standard deviation | | | | | | | | |
| Supplemental Table 9.. Inverse probability of treatment weighting: delayed (>120‐minutes) treatment effects on 30-day mortality in in patients with TIMI flow grade 0-2 | | | | | | | |  |
| **Characteristics** | **Women**  **(N=48)** | | **Men**  **(N=85)** | | **p-value** | | |  |
| Age, years | 69.1 ± 10.5 | | 62.9 ± 10.9 | | 0.001 | | |  |
| **Cardiovascular risk factors, %** |  | |  | |  | | |  |
| Family history of CAD | 20.8 | | 23.5 | | 0.720 | | |  |
| Diabetes | 25.0 | | 23.5 | | 0.851 | | |  |
| Hypertension | 77.1 | | 68.2 | | 0.269 | | |  |
| Hypercholesterolemia | 45.8 | | 44.7 | | 0.901 | | |  |
| Current smoking | 25.0 | | 50.6 | | 0.003 | | |  |
| Former smoking | 8.3 | | 18.8 | | 0.076 | | |  |
| **Previous cardiovascular disease, %** |  | |  | |  | | |  |
| Previous angina pectoris | 22.9 | | 22.4 | | 0.941 | | |  |
| Previous myocardial infarction | 4.2 | | 7.1 | | 0.475 | | |  |
| Previous PCI | 0 | | 1.2 | | 0.320 | | |  |
| Previous CABG | 0 | | 0.4 | | - | | |  |
| Peripheral artery disease | 4.2 | | 7.1 | | 0.475 | | |  |
| Previous heart failure | 6.2 | | 3.5 | | 0.505 | | |  |
| Previous stroke | 4.2 | | 2.4 | | 0.590 | | |  |
| **Clinical presentation at admission** |  | |  | | |  | | |
| ST-segment elevation in anterior leads, % | 47.9 | | 52.9 | | 0.582 | | |  |
| Killip Class ≥ 2, % | 58.3 | | 35.3 | | 0.011 | | |  |
| Systolic blood pressure at baseline, mmHg | 130.0 ± 31.3 | | 132.3 ± 24.2 | | 0.666 | | |  |
| Heart rate at baseline, beats/min | 85.9 ± 21.2 | | 80.7 ± 18.7 | | 0.161 | | |  |
| Serum creatinine at baseline, µmol/liter | 112.9 ± 112.1 | | 115.6 ± 105.5 | | 0.892 | | |  |
| **Outcomes** |  | |  | | |  | | |
| Primary outcome: 30-day mortality, % | 27.1 | | 12.9 | | | 0.061 | | |
| Odds Ratio (95% CI) | 2.48 (0.92 – 6.80) | | | | | 0.059 | | |
| Data are presented as percentages (%). Abbreviations: CABG, coronary-artery bypass grafting; CAD coronary artery disease; CI confidence intervals; OR odds radio; PCI percutaneous coronary intervention; SD, standard deviation | | | | | | | | |
| \| Supplemental Table 10. Inverse probability of treatment weighting: women versus men in patients aged 60 year or over. \| \| \| \| \| --- \| --- \| --- \| --- \| \| **Characteristics** \| **Women**  **(n=462)** \| **Men**  **(n=878)** \| ***P* value** \| \| Age, years \| 69.4 ± 7.5 \| 69.4 ± 7.4 \| 0.908 \| \| **Cardiovascular risk factors, %** \|  \|  \|  \| \| Family history of CAD \| 22.4 \| 22.1 \| 0.900 \| \| Diabetes \| 25.3 \| 25.9 \| 0.811 \| \| Hypertension \| 76.6 \| 74.4 \| 0.376 \| \| Hypercholesterolemia \| 43.3 \| 43.2 \| 0.972 \| \| Current smoking \| 31.7 \| 33.0 \| 0.553 \| \| Former smoking \| 12.3 \| 14.0 \| 0.385 \| \| **Previous cardiovascular disease, %** \|  \|  \|  \| \| Previous angina pectoris \| 21.9 \| 22.7 \| 0.739 \| \| Previous myocardial infarction \| 5.0 \| 7.6 \| 0.071 \| \| Previous PCI \| 2.0 \| 3.9 \| 0.062 \| \| Previous CABG \| 0 \| 0.8 \| 0.047 \| \| Peripheral artery disease \| 1.8 \| 1.6 \| 0.785 \| \| Previous heart failure \| 5.9 \| 6.7 \| 0.571 \| \| Previous stroke \| 3.7 \| 3.3 \| 0.702 \| \| **Clinical presentation at admission, %** \|  \|  \|  \| \| ST-segment elevation in anterior leads \| 38.7 \| 37.2 \| 0.591 \| \| Killip Class ≥2 \| 17.0 \| 16.7 \| 0.889 \| \| Systolic blood pressure at baseline, mmHg \| 136.4 ± 29.2 \| 136.5 ± 25.3 \| 0.936 \| \| Heart rate at baseline, beats/min \| 79.5 ± 18.0 \| 79.4 ± 19.2 \| 0.901 \| \| Serum creatinine at baseline, µmol/liter \| 84.5 ± 25.6 \| 95.2 ± 48.5 \| <0.0001 \| \| **Outcomes** \|  \|  \|  \| \| TIMI flow grade ≤ 2, % \| 12.1 \| 8.1 \| 0.0179 \| \| TIMI flow grade ≤2, OR (95% CI) \| 1.56 (1.08 – 2.26) \| \| 0.0188 \| \| Values are %, mean ± SD or odd ratio (95% confidence intervals)  Abbreviations: CABG, denotes coronary-artery bypass graft; CAD, coronary artery disease; CI confidence interval; OR, odds radio; PCI, percutaneous coronary intervention; SD, standard deviation; TIMI, Thrombolysis In Myocardial Infarction \| \| \| \| | | | | | | |  |  |
| \| Supplemental Table 11. Inverse probability of treatment weighting: women versus men in patients aged < 60 year. \| \| \| \| \| --- \| --- \| --- \| --- \| \| **Characteristics** \| **Women**  **(n=211)** \| **Men**  **(n=1045)** \| ***P* value** \| \| Age, years \| 51.1 ± 6.1 \| 50.9 ± 6.4 \| 0.563 \| \| **Cardiovascular risk factors, %** \|  \|  \|  \| \| Family history of CAD \| 45.3 \| 40.4 \| 0.187 \| \| Diabetes \| 18.0 \| 16.5 \| 0.595 \| \| Hypertension \| 53.2 \| 55.6 \| 0.523 \| \| Hypercholesterolemia \| 45.2 \| 43.9 \| 0.729 \| \| Current smoking \| 70.5 \| 67.1 \| 0.336 \| \| Former smoking \| 7.3 \| 11.3 \| 0.085 \| \| **Previous cardiovascular disease, %** \|  \|  \|  \| \| Previous angina pectoris \| 15.3 \| 16.1 \| 0.773 \| \| Previous myocardial infarction \| 6.4 \| 6.4 \| 1.000 \| \| Previous PCI \| 3.0 \| 3.8 \| 0.574 \| \| Previous CABG \| 0 \| 0.1 \| 0.608 \| \| Peripheral artery disease \| 0 \| 1.0 \| 0.137 \| \| Previous heart failure \| 3.9 \| 3.4 \| 0.719 \| \| Previous stroke \| 2.1 \| 1.5 \| 0.524 \| \| **Clinical presentation at admission, %** \|  \|  \|  \| \| ST-segment elevation in anterior leads \| 36.3 \| 38.0 \| 0.642 \| \| Killip Class ≥2 \| 7.6 \| 7.8 \| 0.921 \| \| Systolic blood pressure at baseline, mmHg \| 134.7 ± 24.2 \| 135.3 ± 23.5 \| 0.741 \| \| Heart rate at baseline, beats/min \| 78.3 ± 15.2 \| 79.3 ± 16.1 \| 0.397 \| \| Serum creatinine at baseline, µmol/liter \| 67.0 ± 14.2 \| 86.9 ± 42.8 \| <0.0001 \| \| **Outcomes** \|  \|  \|  \| \| TIMI flow grade ≤ 2, % \| 2.1 \| 3.7 \| 0.2459 \| \| TIMI flow grade ≤2, OR (95% CI) \| 0.56 (0.21 – 1.51) \| \| 0.2522 \| \| Values are %, mean ± SD or odd ratio (95% confidence intervals)  Abbreviations: CABG, denotes coronary-artery bypass graft; CAD, coronary artery disease; CI confidence interval; OR, odds radio; PCI, percutaneous coronary intervention; SD, standard deviation; TIMI, Thrombolysis In Myocardial Infarction \| \| \| \| | | | | | | |  |  |

# REFERENCES:

1. Troyanskaya O, Cantor M, Sherlock G, et al. Missing value estimation methods for DNA microarrays. Bioinformatics. 2001;17:520-5.
2. Beretta L, Santaniello A. Nearest neighbor imputation algorithms: a critical evaluation. BMC Med Inform Decis Mak. 2016;16 Suppl 3:74.
3. Kononenko I, Simec E, Robnik-Sikonja M. Overcoming the myopia of inductive learning algorithms with RELIEFF. Appl Intell. 1997;7:39-55.
